# Streptophyte phytochromes exhibit an N-terminus of cyanobacterial origin and a C-terminus of proteobacterial origin

**DOI:** 10.1186/s13104-015-1082-3

**Published:** 2015-04-13

**Authors:** Thorsten Buchberger, Tilman Lamparter

**Affiliations:** Karlsruhe Institute of Technology (KIT), Botanical Institute, Kaiserstr. 2, Karlsruhe, D-76128 Germany

**Keywords:** Bacteria, Cyanobacteria, Fungi, Gene transfer, Histidine kinase, Phytochrome, Plants

## Abstract

**Background:**

Phytochromes are red light-sensitive photoreceptors that control a variety of developmental processes in plants, algae, bacteria and fungi. Prototypical phytochromes exhibit an N-terminal tridomain (PGP) consisting of PAS, GAF and PHY domains and a C-terminal histidine kinase (HK).

**Results:**

The mode of evolution of streptophyte, fungal and diatom phytochromes from bacteria is analyzed using two programs for sequence alignment and six programs for tree construction. Our results suggest that Bacteroidetes present the most ancient types of phytochromes. We found many examples of lateral gene transfer and rearrangements of PGP and HK sequences. The PGP and HK of streptophyte phytochromes seem to have different origins. In the most likely scenario, PGP was inherited from cyanobacteria, whereas the C-terminal portion originated from a proteobacterial protein with multiple PAS domains and a C-terminal HK. The plant PhyA and PhyB lineages go back to an early gene duplication event before the diversification of streptophytes. Fungal and diatom PGPs could have a common prokaryotic origin within proteobacteria. Early gene duplication is also obvious in fungal phytochromes.

**Conclusions:**

The dominant question of the origin of plant phytochromes is difficult to tackle because the patterns differ among phylogenetic trees. We could partially overcome this problem by combining several alignment and tree construction algorithms and comparing many trees. A rearrangement of PGP and HK can directly explain the insertion of the two PAS domains by which streptophyte phytochromes are distinguished from all other phytochromes.

**Electronic supplementary material:**

The online version of this article (doi:10.1186/s13104-015-1082-3) contains supplementary material, which is available to authorized users.

## Background

Phytochromes are photoreceptors with a bilin chromophore that are found in plants, bacteria and fungi but missing in animals and Archaea. In plants, they act as the dominant photoreceptors affecting most developmental processes, from stimulation of seed germination over de-etiolation and shade avoidance to flowering [1]. Phytochromes are often lacking in algae, but they have been found in the Zygnematales *Mougeotia scalaris* and *Mesotaenium caldariorum* [2], diatoms [3], brown algae, Prasinophytes and glaucophytes [4]. In fungi, phytochromes regulate sporulation and the transition from sexual to asexual development [5]. Only a few effects of phytochromes have been reported in bacteria: in species of the genera *Bradyrhizobium* and *Rhodopseudomonas*, phytochromes control the synthesis of photosynthetic pigments [6,7], and in *Azospirillum brasilense*, bacteriophytochrome modulates the stress response [8]. The role of phytochromes in cyanobacteria remains unclear [9]. However, cyanobacteria also contain biliproteins that are partially homologous to phytochromes, which are termed cyanobacteriochromes [10] and modulate various effects, including phototaxis [11,12] and chromatic adaptation [13-15].

Phytochromes present two spectrally distinct long-lived forms, termed Pr and Pfr, that serve as red-absorbing and far-red absorbing forms, respectively [16]. A few bacterial phytochromes [17,18], a few algal phytochromes [4] and most cyanobacteriochromes [15,19-21] absorb in other spectral ranges but still retain two spectrally distinct long-lived forms. In classical phytochromes, light triggers both Pr to Pfr and Pfr to Pr photoconversion. Red light establishes a high Pfr level, whereas far-red light produces mainly Pr. The Pr form is synthesized in darkness, and this form is typically stable in the dark. Pfr is either stable or undergoes slow dark conversion to Pr. Some bacterial phytochromes termed “bathy-phytochromes” convert from Pr to Pfr in darkness [7,22,23], these phytochromes therefore have a Pfr dark state.

**Figure 1:**
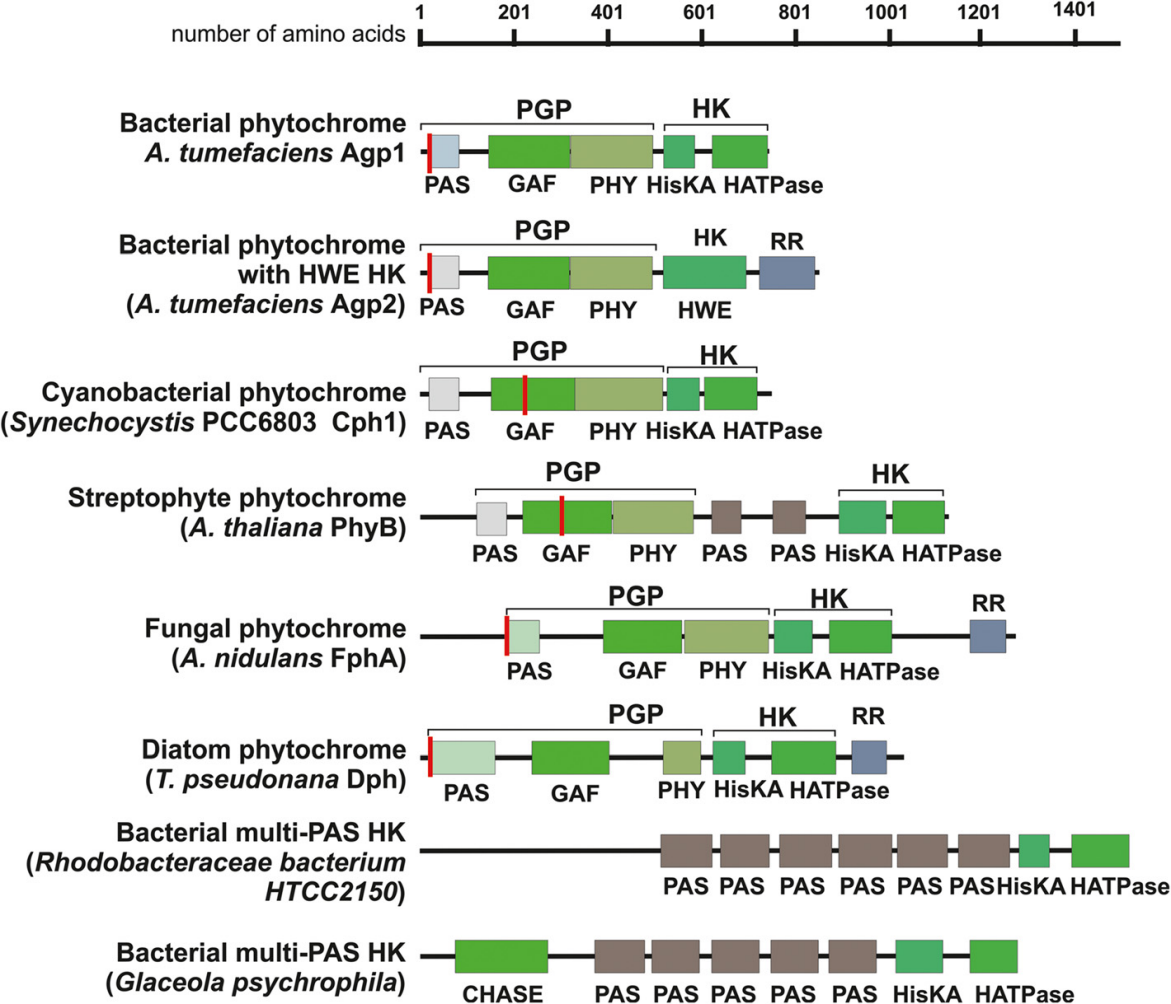
Domain arrangements of phytochromes and bacterial multi-PAS histidine kinases. The PFAM [79] domain names are given under each bar; the ranges of PGP and HK are indicated above the bars; RR, response regulator. The position of the chromophore-binding Cys residue is indicated by a vertical red bar. Accession numbers for phytochromes are given in Additional file 3.

Phytochromes are multidomain proteins that carry an N-terminal tridomain referred to as a PGP domain, which consists of a PAS domain [24], followed by a GAF domain [25] and a PHY domain [26,27] (Figure 1). The chromophore-binding Cys residue of fungal and most bacterial phytochromes lies in the N-terminus of the PAS domain [28,29]. The presence of this cysteine correlates with the incorporation of biliverdin (BV) as a chromophore [30,31]. Certain cyanobacterial phytochromes of the “CphB” type use the same chromophore [32] and the same binding site. All other cyanobacterial phytochromes belong to the “CphA” type, which incorporate phycocyanobilin (PCB) as a chromophore [33]. The chromophore-binding Cys residue lies within the GAF domain of the protein. Plant phytochromes employ the same binding site but carry a less reduced chromophore termed phytochromobilin (PΦB) [34]. The cyanobacteriochromes exhibit one or more GAF domains for chromophore insertion and a cysteine for covalent binding. The GAF domain(s) can be combined with various other domains [10]; in certain cases, a PHY domain is placed at the C-terminal end of the GAF domain.

Domain rearrangement, a central principle in evolution, has occurred frequently within the phytochrome family. In some proteins, a PYP domain is fused to the N-terminus of bacterial phytochromes [18,27]. A few bacterial phytochromes contain a markedly short C-terminal sequence in addition to the PGP domain [7], and there are also examples of phytochromes with a C-terminal GGDEAF domain [35]. The prototypical phytochrome exhibits a C-terminal histidine kinase (HK) comprising an ATP-binding/ATPase domain (HATPase) at its C-terminus and a dimerization and substrate domain (HisKA) that contains the His residue phosphorylated by the HATPase domain [26]. Land plants and the Zygnematales green algae *Mougeotia* and *Mesotaenium* show the same phytochrome domain arrangement, indicating a close relationship between the two groups. These phytochromes carry both HATPase and HisKA domains, but (with the exception of monocotyledonous PhyA) the substrate His residue is replaced by an Arg or Glu. The kinase activity was apparently lost during evolution. (This region is nevertheless also abbreviated as HK here.) Between the HK and the PGP of streptophyte phytochromes, two PAS domains are observed that are not present in other phytochromes (Figure 1). Prasinophyte, glaucophyte and heterokont phytochromes exhibit a HK with conserved substrate His and sometimes an additional response regulator (RR) at its C-terminus. There is no double PAS-domain between PGP and HK in these cases, but prasinophyte phytochromes can contain one PAS domain. Certain bacterial phytochromes display a HK type that is distinct from all others, termed HWE-His kinase, according to the amino acids that are specifically conserved in this group [36]. These phytochromes also present an additional C-terminal response regulator (RR), whereas the cognate response regulators of other bacterial phytochromes are expressed as separate proteins. Many members of the phytochromes with HWE-HKs and a C-terminal RR belong to the bathy phytochromes [23]. Fungal and diatom phytochromes also carry a C-terminal RR (Figure 1), but their HK is of the classical type [3,29,37]. The two fungal phytochromes that have been studied biochemically exhibit a Pr ground state [29,37,38].

Other rearrangements occurred during the evolution of streptophyte phytochromes. “Neochromes” are fusions of an N-terminal PGP with a C-terminal phototropin, which is a blue light sensor with two FMN chromophores. This type of photoreceptor has been found in Zygnematales, algae, hornworts and ferns, in which the same type of rearrangement has occurred several times [39-41]. Another rearrangement was found for phytochrome 1 of the moss *Ceratodon purpureus*. In this phytochrome, the N-terminal PGP domain is fused with a C-terminal serine/threonine protein kinase [42].

Despite the present wealth of available sequence information, it remains unclear how eukaryotic phytochrome genes arose out of prokaryotic sequences. Typical cyanobacterial, prasinophyte and streptophyte phytochromes share common properties: their PCB and PϕB chromophores differ from BV based on the presence of the reduced ring A double bond and they share a chromophore-binding cysteine, which lies within the GAF domain. Therefore, prasinophyte and streptophyte phytochromes might have arisen from the cyanobacterial endosymbiont that gave rise to the plastids of photosynthetic eukaryotes. However, phylogenetic studies in which the origin of streptophyte phytochromes is addressed are not reliable. Firstly, bootstrap values [43] are always low at the suggested transition points from prokaryotes to eukaryotes. Secondly, a close relationship between plant and cyanobacterial phytochromes has been observed in published phylogenetic studies [23,44], whereas other studies imply a distinct prokaryotic origin of streptophyte phytochromes [31,45]. The prokaryotic origin of fungal phytochromes is also uncertain, although their origin is clearly different from that of streptophytes.

There is also uncertainty regarding the times at which the major angiosperm phytochrome lineages diverged. Based on homology and function, angiosperm phytochromes are referred to as PhyA to PhyE according to the founding members of *Arabidopsis thaliana*. PhyA and PhyC are closely related, as are PhyB, D and E. PhyD arose later following a gene duplication event and is found exclusively in the *Brassicaceae* family [46]. PhyF is the PhyC homolog of tomato [47] and is not found in other species. PhyA is highly concentrated in dark-grown seedlings, allowing early protein purification and biochemical studies [48,49]. This member differs from the other phytochromes in its high sensitivity. The effects of PhyA are induced by markedly weak light or by far-red light, and PhyA mediates the so-called high irradiance response, which can be induced by continuous far-red light [50-52]. Most studies on the specific properties of PhyA have been performed in angiosperms, but far-red high-irradiance responses are also observed in gymnosperms [53] and mosses [54]. Outside of the angiosperms, these specific features are not necessarily linked to PhyA homologs, but phylogenetic studies have shown that the separation of the type A and type B lineages occurred before the angiosperms arose [55].

The problem of uncertain eukaryote/prokaryote branching points is a factor in all approaches involving the construction of phylogenetic trees using a single program. A reasonable number of algorithms and computer programs are available for multiple alignments and tree constructions [56,57]. We therefore performed phylogenetic analyses using a combination of various programs. All of the obtained trees together provided a clearer picture of the early evolution of prokaryotic and eukaryotic phytochromes. Streptophyte phytochromes have most likely arisen from a fusion of a cyanobacterial PGP with a PAS/PAS/HK from a non-cyanobacterial prokaryote. The divergence between the PhyA and PhyB lineages must be placed during the early evolution of streptophytes. Similarly, early phytochrome gene duplication occurred during the evolution of fungi. This study also revealed several examples of lateral gene transfer of phytochromes within bacteria and provides evidence of HK replacements of bacterial phytochromes.

## Results and discussion

**Figure 2:**
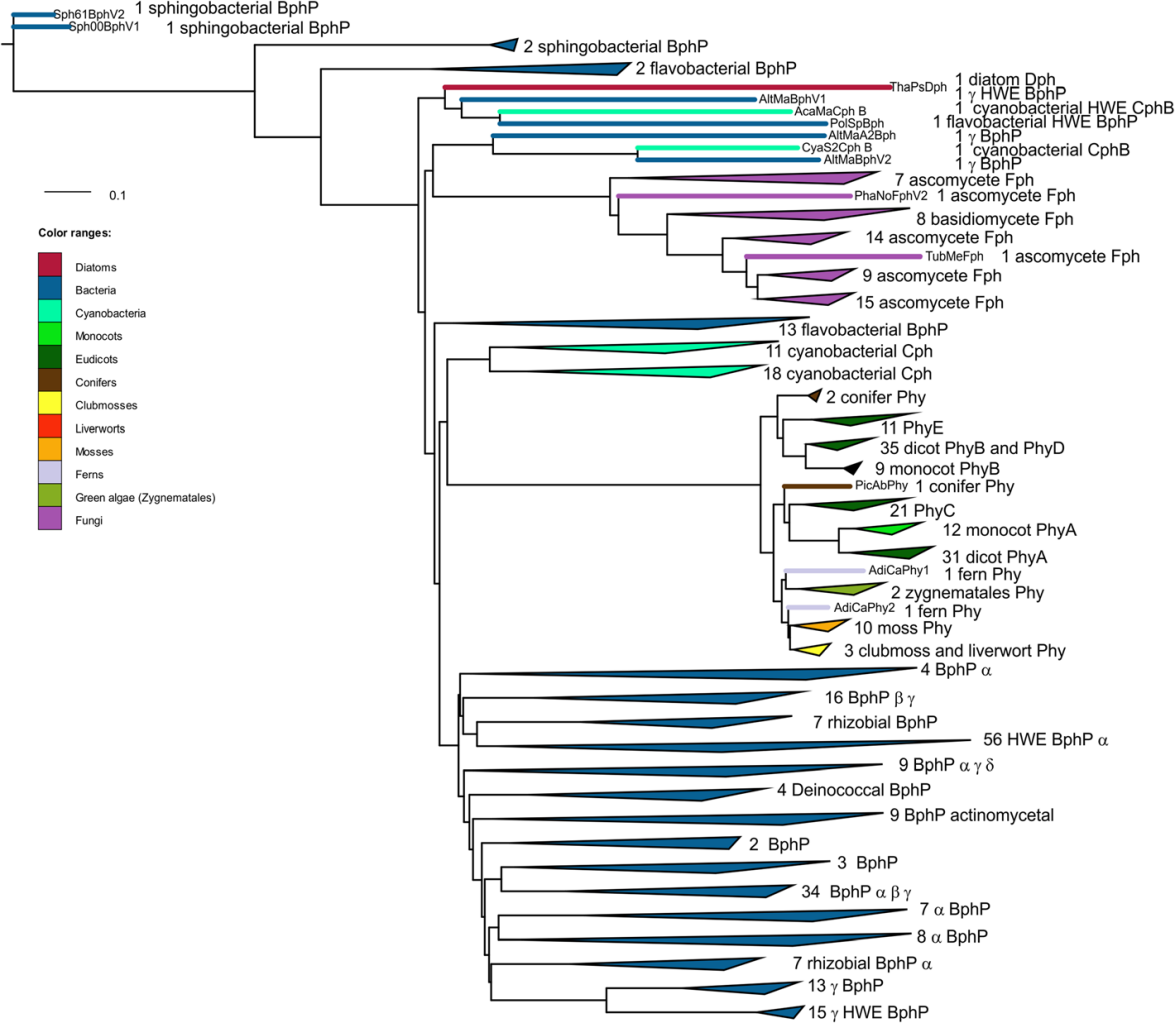
Example for a phylogenetic PGP tree with clades presented in collapsed mode. See Additional file 1 for all PGP trees and Additional file 4 for a tree summary. Sequences were aligned with MUSCLE, the tree was constructed with PhyML using the WAG matrix (tree “P14”). BphP, Phy, Cph and Fph stands for bacterial, streptophyte, cyanobacterial or fungal phytochrome, respectively. The Greek letters stand for proteobacterial subgroups. If this letter is printed in front of the abbreviation for phytochrome, all members of that clade are in the same subgroup, if printed thereafter, only the majority belongs to that subgroup.

**Figure 3:**
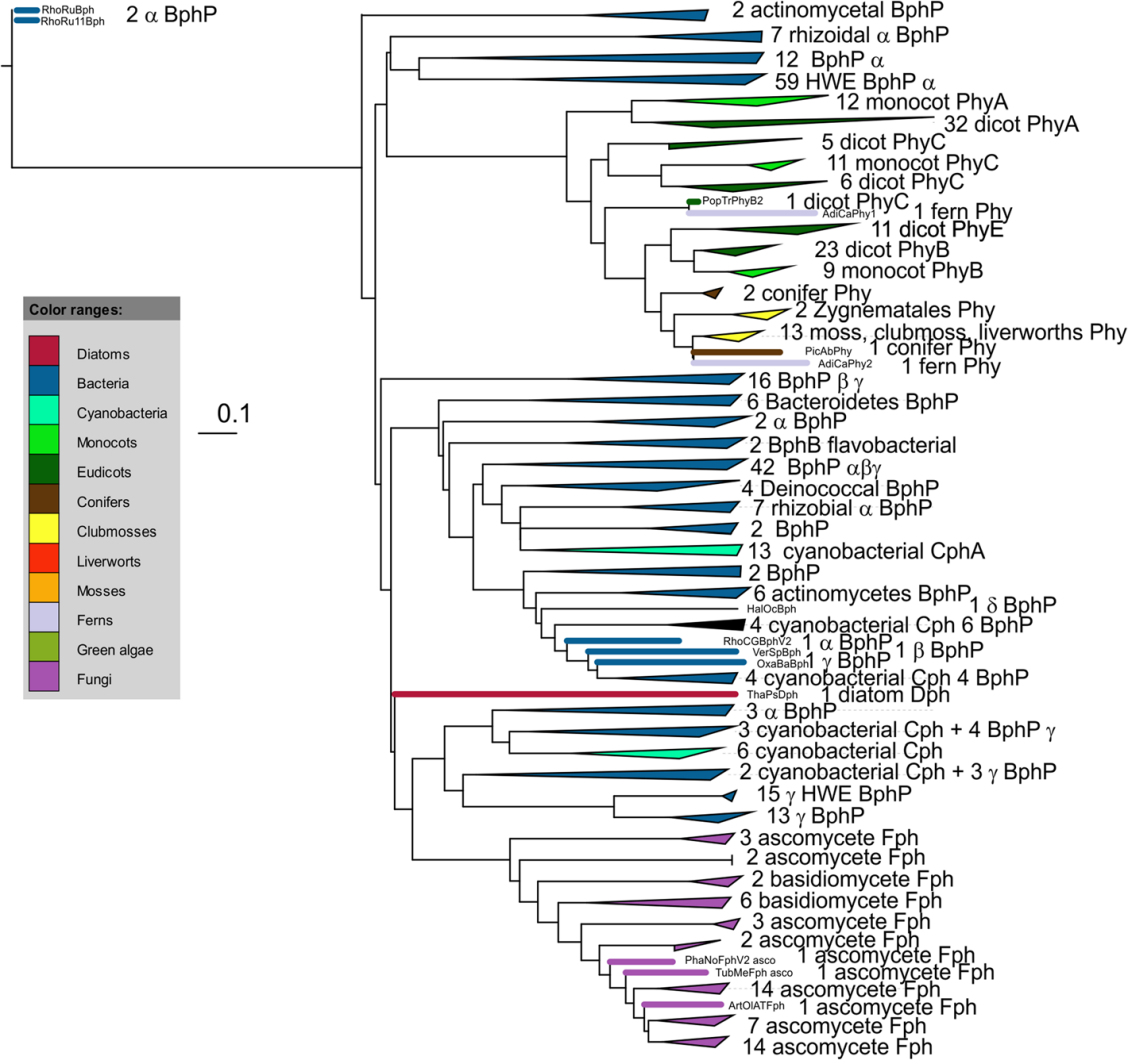
Example for a phylogenetic HK tree. See Additional file 2 for all HK trees and Additional file 5 for a tree summary. Sequences were aligned with MUSCLE, the tree was constructed with PhyML using the WAG matrix (tree “H10”). BphP, Phy, Cph and Fph stands for bacterial, streptophyte, cyanobacterial or fungal phytochrome, respectively. The Greek letters stand for proteobacterial subgroups. If this letter is printed in front of the abbreviation for phytochrome, all members of that clade are in the same subgroup, if printed thereafter, only the majority belongs to that subgroup.

The present approach is based on the construction of multiple trees using two alignment programs, six phylogenetic programs and variations of the replacement matrices. We selected only proteins with an N-terminal PGP tridomain and a C-terminal HK, which is the most common motif of bacterial and eukaryotic phytochromes. As in earlier studies, alignments and tree constructions were performed separately for PGP and HK [31]. Thus, rearrangements between N- and C-terminal moieties can be recognized. Streptophyte phytochromes exhibit an insertion between PGP and HK, and the present study led to a possible explanation for how this specialty arose (see below). Figures 2 and 3 show PGP and HK trees, respectively, with collapsed clades. The collection of all PGP and HK trees is shown in Additional files 1 and 2.

### Bacteria

As expected, the sequences of phylogenetically related bacteria clustered together. However, the sequences of larger groups, such as the α-, ß- or γ-proteobacteria, are split between several subgroups located at various positions within the trees, and single outliers are often found within groups of unrelated bacteria. Both observations indicate that lateral transfer, a major principle in the evolution of prokaryotes [58], has occurred frequently during the evolution of bacterial phytochromes. Additionally, there is evidence of rearrangements of N- and C-terminal sequences, as the branching patterns in PGP trees often vary from those in the HK trees. For example, the PGPs of Agp1 from the α-proteobacterium *Agrobacterium tumefaciens* strains C58 and F2 (AgrTuAgp1/AgrTuFAgp1) are always grouped with three sequences from the genus *Methylobacteria* (e.g., MetSpBphV1); in the HK trees, Agp1 sequences are grouped with *Phenylobacterium zucineum* HLK1 (AzoAmBph/PheZuBph) and *Azospirillum amazonense* Y2 (Additional files 1 and 2).

The trees provided an interesting suggestion regarding the origin of phytochrome: in all PGP trees, the phytochromes at the base, i.e., those that are most distantly related to all others, belong to *Flavobacteriaceae*, *Chryseobacterium gleum* or *Sphingobacterium spiritivorum*, members of the phylum Bacteroidetes (Table 1). The PGP domain of the cyanobacterium *Acariochloris marina* is also often found close to the base, closely related to a *Polaribacter* (a flavobacterium) PGP. The *A. marina* PGP was gained via horizontal gene transfer (see below). The results could indicate that phytochromes evolved in ancestors of Bacteroidetes. This group contains water-dwelling species, a few cold-tolerant species, and a few pathogenic species; bacteriochlorophyll-based photosynthesis is not observed in this group.

**Table 1:** **Groups found at the base of PGP or HK trees**

	**PGP trees (total of 17) number of trees**	**HK trees (total of 13) number of trees**
Sphingobacteria (phylum Bacteroidetes) at base	5	0
Flavobacteria (phylum Bacteroidetes) at base	11	0
HWE histidin kinases at base	0	9
*Rnhodospirillum* HK at base	0	4
*Polaribacter* (phylum Bacteroidetes) at the base of HWE HK	0	9

For further details see Additional files 1, 2, 4 and 5.

### Cyanobacteria

Non-cyanobacterial bacteria and cyanobacterial phytochromes of the CphB type utilize BV as a chromophore, which is synthesized by heme oxygenase from heme. The CphA-type phytochromes of cyanobacteria use PCB as a chromophore, which is synthesized from BV by the biliverdin reductase PCYA [59,60]. The change of the chromophore in cyanobacterial CphA-type phytochromes results in a spectral blue shift to approximately 655 nm for the Pr maximum [61], which is closer to the absorption maxima of their photosynthetic pigments than the absorption maximum of BV-binding phytochromes (approximately 700 nm) [28]. With the chromophore-binding site of CphA-type phytochromes in the GAF domain, the protein becomes independent of the PAS domain, paving the way for the evolution of cyanobacteriochromes that lack the PAS domain [10]. The origin of phytochrome outside of the cyanobacteria suggests that phytochrome evolved earlier than did cyanobacteria. The formation of BV by heme oxygenase requires oxygen as a second substrate [62], which was not abundant in the atmosphere before cyanobacteria evolved. We speculate that heme synthesis and heme oxygenase evolved in a low-oxygen environment or that the enzyme utilizes oxygen produced via cellular metabolism.

In 6 of 17 PGP and in 6 of 13 HK trees, the homogeneous group of cyanobacteria contains a sequence of *Oscillochloris trichoides*, which belongs to the Chloroflexi or green non-sulfur bacteria (Table 2). The phytochrome of *O. trichoides* could therefore have been transferred from a cyanobacterium by lateral gene transfer. BLAST searches indicate that the *O. trichoides* genome is the only one out of 50 sequenced Chloroflexi genomes that contains a phytochrome gene. This phytochrome shows the characteristic N-terminal Cys for BV chromophore incorporation.

**Table 2:** **The** ***Oscillochloris trichoides*** **sequence is often found among cyanobacterial sequences**

	**PGP trees (total of 17) number of trees**	**HK trees (total of 13) number of trees**
*Oscillochloris trichoides* phytochrome among cyanoabacteria	6	6

In addition, there is clear evidence of gene transfers from non-cyanobacterial bacteria to cyanobacteria (Table 3). The above-mentioned species *Acariochloris marina* is a marine cyanobacterium that has drawn interest because it synthesizes chlorophyll d instead of chlorophyll a as its major photosynthesis pigment [63,64]. A conserved cysteine in the N-terminus of this phytochrome again indicates the use of BV as a chromophore. In the PGP trees, *A. marina* always forms a group with the flavobacterium *Polaribacter* sp. MED152 and is also often found at the base of the trees. In the HK trees, the *A. marina* sequence is placed among other HWE-HKs, again, often in a group with *Polaribacter* or in close vicinity to it (Table 4). Thus, the *A. marina* phytochrome arose from a lateral gene transfer event from flavobacterium to cyanobacterium. This gene transfer could have occurred quite early in the evolution of cyanobacteria.

**Table 3:** **Phytochromes of 2 or 3 cyanobacteria are separate from the other cyanobacterial species, evidence for horizontal gene transfer**

	**PGP trees (total of 17) number of trees**	**HK trees (total of 13) number of trees**
*Acariochloris marina* outside cyanobacteria	17	13
*Cyanothece* sp. CCY0110 outside cyanobacteria	17	13
*Microcoleus vaginatus* FGP-2 outside cyanobacteria	0	12

**Table 4:** **Phytochromes of** ***Acariochloris marina*** **and** ***Polaribacter*** **sp. MED152 are often closely related**

	**PGP trees (total of 17) number of trees**	**HK trees (total of 13) number of trees**
*Acariochloris marina* and *Polaribacter* sp. MED152 next to each other	15	9

*Cyanothece* sp. CCY0110 is another marine cyanobacterium that obtained a phytochrome through lateral gene transfer (Table 3); this protein also shows a BV-chromophore signature. Six other *Cyanothece* sequences are found within the main group of cyanobacterial sequences and exhibit the PCB chromophore signature.

In all HK trees, the cyanobacterial sequences are split into 3 to 5 major groups. This difference in the overall distribution of cyanobacteria between the N- and C-terminal sequences indicates rearrangements between PGPs and HKs. The exchange of the kinase, likely coupled to the exchange of the cognate response regulator, which is often encoded within the same operon downstream of the kinase-encoding sequence [65], allows easy switching between various signal transduction cascades.

### HWE His kinases

Phytochromes that carry HKs of the HWE type also exhibit a C-terminal response regulator domain [23,36]. Because phytochromes with an HWE-HK are most abundant in rhizobia, it has been suggested that such phytochromes evolved in this group [23]. The basal position of HWE-HKs in the present study (Table 1) suggests that the first phytochromes presented a C-terminal HWE-HK. Proteobacteria might have inherited an HWE type of phytochrome rather early via lateral gene transfer. No HWE-HK phytochrome is found outside Bacteroidetes and proteobacteria (with the exception of the *A. marina* phytochrome).

Unexpectedly, the HWE-HKs of *Pseudomonas syringae* strains form a group that always remains separate from the major HWE-HK group. All other phytochromes of *P. syringae* appear within a sister group of the HWE-HKs. This split is observed in both HK and PGP trees. The *P. syringae* HWE-HKs are hybrids of an N-terminal domain with an HWE signature [36] and a standard C-terminal ATPase (Additional file 3), which is not recognized in other HWE-HKs. The response regulator domain found in all phytochromes with an HWE-HK, but not in other bacterial phytochromes, is also present in the *P. syringae* phytochromes with an HWE signature and absent in the others. In phytochromes two types of HWE-HKs can be distinguished, one of which specifically evolved in *P. syringae*.

### Eukaryotes

There are three eukaryotic lineages with phytochromes included in the present study: fungi, heterokonts and streptophytes. Streptophytes encompass land plants and their green algal sister group, the Charophytes [66], here represented by two algae of the order Zygnematales. Streptophyte sequences cluster together as one monophyletic group in all species, and the same result was obtained for fungal sequences. Recent sequencing efforts have shown that Prasinophytes, which are green algae that do not belong to the streptophytes, can also contain phytochrome. These sequences are not included here because they were not yet accessible when we initiated our analyses. In subsequent control studies, we constructed NJ and ML trees with and without the PGP of the Prasinophyte *Tetraselmis astigmatica*. The major conclusions regarding the origin of streptophyte PGPs were not affected. Heterokonts are represented here by one phytochrome from the diatom *Thalassiosira pseudonana*.

### Eukaryotes, PGP trees

**Figure 4:**
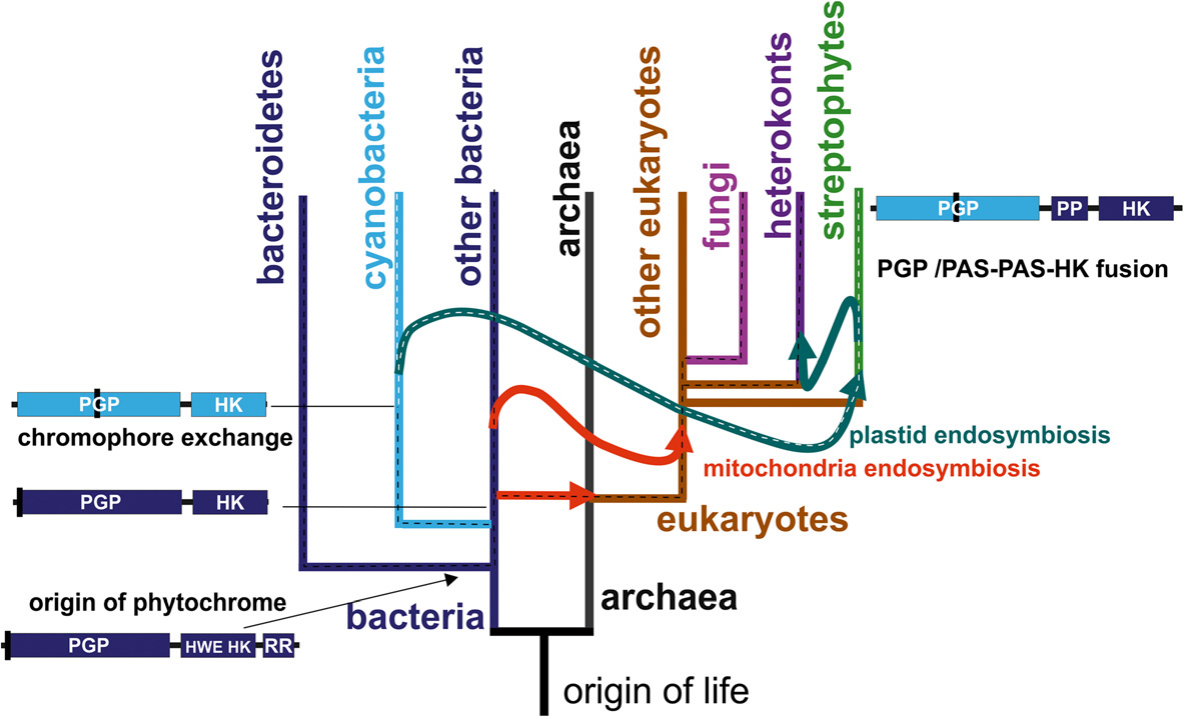
Cartoon representation of the evolution of major groups that contain phytochromes and the pathways of the origin of streptophyte phytochromes. The tree is based on the assumption that eukaryotes evolved from archaea combined with a massive gene transfer from bacteria, as indicated by the straight arrow, to explain the chimeric character of eukaryotes [80]. Endosymbiosis is indicated with curved arrows. The dashed black lines indicate the evolutionary paths of bacterial phytochromes, the dashed white line shows the path of cyanobacterial and plant PGPs. Phytochrome drawings on the left and right side indicate the overall domain arrangement (PP, PAS-PAS domain of streptophyte phytochromes), chromophore-binding position (black line) and bacterial or cyanobacterial origin (blue or light blue line, respectively).

In eight out of 17 PGP trees, streptophyte PGPs appear as a sister group of cyanobacterial PGPs (Table 5), in two of these trees with two or 15 bacterial sequences between them. In four trees, streptophyte PGPs appear as sister group of a group of Bacteroidetes PGPs. In five trees, they appear to have a basal origin, but these branching patterns varied significantly from each other. The close relationship between streptophyte and cyanobacterial PGPs found in about half of all trees points to the most likely origin of plant PGPs as the cyanobacterial endosymbiont that gave rise to the plastids (see Figure 4).

**Table 5:** **Relationship between streptophytes and cyanobacteria**

	**PGP trees (total of 17) number of trees**	**HK trees (total of 13) number of trees**
Streptophytes sister group of cyanobacteria	8	0
Streptophyte sister group of Bacteroidetes	4	0
Streptophyte appear as basal group	5	2
Streptophyte sister of HWE HK	0	3

In 10 of the 17 PGP trees, fungal phytochromes are inserted next to the PGP of *Cyanothece* sp. CCY0110 and its relatives from the γ-proteobacterium *Alteromonas macleodii*, which are sometimes combined with certain other bacteria (Table 6). In three trees, fungal PGPs appear as a sister group of the diatom PGP (Table 6), and in another two trees, the diatom and fungal groups are close together, with few bacteria between them (Additional file 4).

**Table 6:** **Nearest neighbors of fungal phytochromes**

	**PGP trees (total of 17) number of trees**	**HK trees (total of 13) number of trees**
Fungi sister group of Cyanothece sp. CCY0110 and *Alteromonas macleodii*	10	0
Fungi sister group of diatom	3	4
Fungi appear as basal group	3	6

In nine trees, the diatom PGP is placed next to the PGP of *A. marina* and its relatives from *Alteromonas macleodii* and *Polaribacter* sp. In seven trees, this sequence is inserted at a basal position in the tree, and in three trees, it is located close to fungi (Additional file 4). Diatoms arose from a non-photosynthetic eukaryotic cell that incorporated a photosynthetic eukaryotic cell during secondary endosymbiosis [67].

The bacterial phytochromes located closest to the fungal and diatom PGPs are from γ-proteobacteria and from the cyanobacteria *Cyanothece* and *A. marina*, respectively. A close relationship of fungal and diatom PGPs is also evident. It therefore appears that the PGPs of fungi and diatoms arose from the same bacterial progenitor. A mitochondrial origin of the fungal and diatom PGPs is unlikely because a close relationship to α-proteobacteria was not found.

### Eukaryotes, HK trees

With respect to the basal branch point of streptophytes, the HK trees differ significantly from the PGP trees. In the HK trees a close relationship between cyanobacteria and streptophytes was never found. They appear either as sister group of HWE-HKs, or as sister group of fungi or are found at the base of the trees (Table 5). We assume that the C-terminus of streptophyte phytochromes was not inherited via plastidic endosymbiosis and that these phytochromes arose from a fusion of the PGP and HK domains during early streptophyte evolution. The wide diversity regarding the origin of streptophyte HKs can be explained by the assumption that a fusion occurred with a non-phytochrome HK. This topic is addressed below.

The pattern of insertion of fungal HKs is also diverse. In three trees, fungal HKs are placed next to 8 to 10 cyanobacteria and five to 36 other bacteria and in four trees, fungi are close to the diatoms (Table 6, Additional files 2 and 5). Although a common prokaryotic origin of all eukaryotic phytochrome HKs could be speculated, the specific domain arrangement of the streptophyte C-terminal portion, with two PAS insertions between PGP and HK, points to a separate origin for this group. Fungal and diatom phytochromes exhibit the same domain arrangement and could be descendants of the same bacterial protein. The presence of the C-terminal response regulator found in bacterial HWE-HKs, in fungal and diatom phytochromes suggests that fungal and diatom HKs were inherited from bacterial HWE-HKs that had lost their specific HWE features. A rearrangement of the N- and C-termini is also proposed for fungal phytochromes, although evidence of this rearrangement is less solid than for streptophytes.

### The PAS/PAS/HK C-terminus of streptophyte phytochromes

As streptophyte PGPs are most likely of cyanobacterial origin, whereas the HK of streptophytes was not inherited from cyanobacteria, a rearrangement of the N- and C-terminal portions of streptophyte phytochromes must have occurred before diversification into Charophytes and land plants. As discussed above, similar rearrangements have occurred frequently within bacteria. Histidine kinases are important signaling proteins in prokaryotes; there are far more HKs than phytochromes found in the databases. The PGP of streptophyte phytochromes was therefore most likely rearranged with an HK that was previously not part of a phytochrome. When we performed BLAST searches using the HK sequence from the moss *Ceratodon purpureus* phytochrome 2 as an input and restricted the search to prokaryotes, there were indeed only two phytochromes among the first 1000 hits. Since we found several proteins with a PAS/PAS/HK motif (see cartoons Figure 1 below as example), we performed subsequent searches using the entire PAS/PAS/HK sequence of *C. purpureus* phytochrome and identified 38 bacterial proteins (among the first 500 hits) and 41 archaeal proteins with a PAS/PAS/HK pattern.

To determine whether the plant PAS/PAS/HK is of archaeal or bacterial origin, we constructed again phylogenetic trees using five distinct programs. Most of the archaeal sequences form one or two major groups, and most of the bacterial sequences form another group containing few archaeal outliers. In three trees, both streptophyte PAS/PAS/HK representatives included in the present analysis are located at the base of the tree, as sisters of most bacterial and archaeal members. In one tree, both streptophyte sequences appear as a sister group of two bacterial sequences (See Figure 1 for the domain arrangements). In another tree, streptophytes are positioned as a sister group of the same two bacterial and two archaeal (*Methanoplanus limicola* and *Methanoregula formicica SMSP*) sequences (see Additional File 6 for trees). These results favor a proteobacterial origin of the PAS/PAS/HK of streptophyte phytochromes. Together with the close relationship between streptophyte and rhizobial HK subunits described above, it is possible that the PAS/PAS/HK of streptophytes might have originated from the mitochondrial endosymbiont. Repeating this study with a greater number of sequences in the future will provide a clearer picture of the origin of the streptophyte phytochrome C-terminus.

### Origin of streptophyte phytochromes

**Figure 5:**
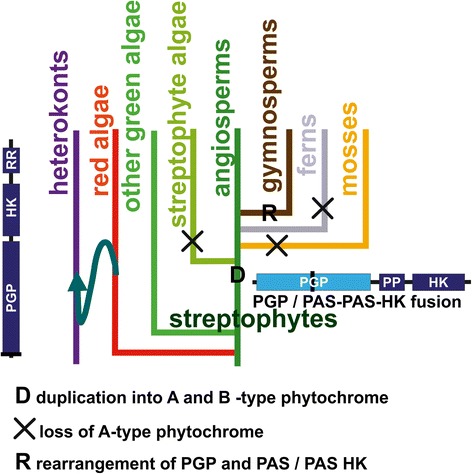
Cartoon representation of major events during the evolution of streptophyte phytochromes. D indicates the duplication of phytochrome genes that gave rise to A- and B-type phytochromes. X indicates loss of A-type phytochromes; R indicates the rearrangement of PGP and PAS/PAS/HK in the evolution of gymnosperm P-type phytochromes. Secondary endosymbiosis is indicated by the curved arrow. The overall domain arrangement of diatom (heterokont, left) and streptophyte phytochrome is also shown.

Regarding the early evolution of streptophyte phytochromes, the following picture emerges. At the time when the cyanobacterial endosymbiont was engulfed by the mother cell, the PAS/PAS/HK gene sequence was already present in the nuclear genome. The cyanobacterial PGP gene moved from the plastid to the nucleus and was fused with the PAS/PAS/HK gene to give rise to the current streptophyte phytochrome genes. The advantage of the cyanobacterial PGP domain lies in the lower wavelength absorption maximum of PCB-binding vs. BV-binding phytochromes (approximately 655 nm vs. 700 nm for the Pfr forms), which is better suited to sensing chlorophyll a. The original chromophore, PCB, was then replaced by PΦB, leading to a further tuning of the Pr absorption maximum to 665 nm. Cartoons depicting the evolution of eukaryotic and streptophyte phytochromes are in Figures 4 and 5.

### Evolution of angiosperm phytochromes A and B

The present phylogenetic trees also provide insight into the evolution of various types of streptophyte phytochromes. Every species exhibits several phytochromes, which can be distinguished by an additional letter or an additional number. The grouping of PhyA/C/F (A-type phytochromes) and PhyB/D/E (B-type phytochromes) is reflected in all trees of the present study. All cryptogam (moss, clubmoss, fern and green algal) phytochromes are unified in one branch. In all PGP and HK trees with one exception, the cryptogam group is positioned within the A-type lineage or within the B-type lineage (Table 7). The branching of seed plant and from cryptogam phytochromes can therefore not clearly be assigned. This pattern suggests nevertheless that the diversification into A- and B-type phytochromes dates to the time around the diversification of green algae and plants, i.e., earlier than predicted previously [55]. The early duplication of phytochrome genes could be related to a genome duplication event that provided the basis for an increase in gene numbers associated with early streptophyte evolution.

**Table 7:** **Position of cryptogam phytochromes**

	**PGP trees (total of 17) number of trees**	**HK trees (total of 13) number of trees**
Cryptogams within PhyA branch	8	1
Cryptogams within PhyB branch	8	12

### Two lineages of fungal phytochromes

**Figure 6:**
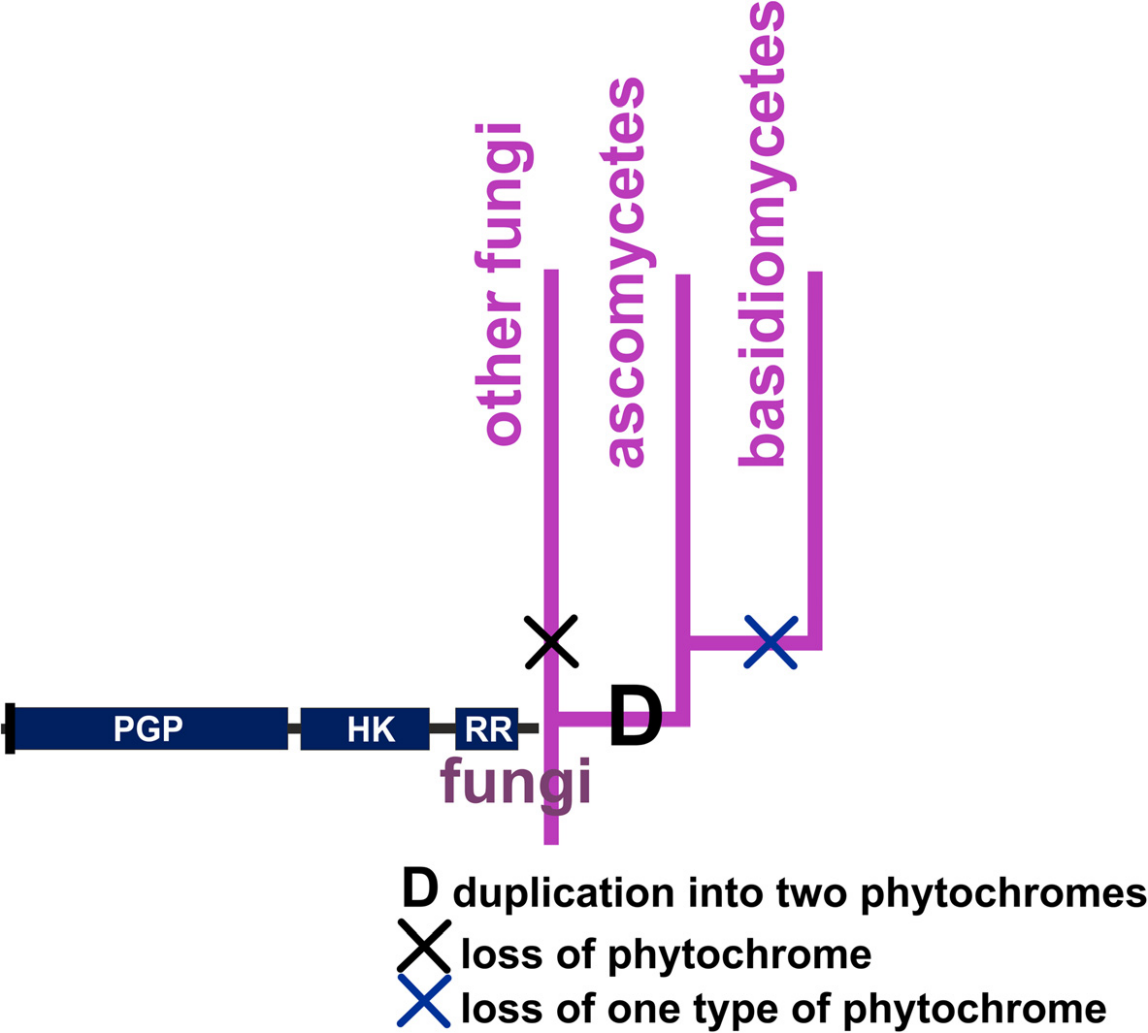
Diagram of the evolutionary pathway of fungal phytochromes. D indicates gene duplication; X indicates loss of one type of phytochrome (in the basidiomycetes) or all phytochromes (in “other fungi”).

All known sequences of fungal phytochromes belong to the phyla Ascomycetes and Basidiomycetes; no phytochromes were identified in other fungal phyla in the present work. Although Ascomycetes and Basidiomycetes are separate monophyletic groups, Basidiomycete sequences appeared most often as a subgroup of Ascomycetes (Table 8). The fungal phytochromes generally form five subgroups, one with Basidiomycete and four with Ascomycete sequences (Additional files 4 and 5). In typical PGP trees, the basal subgroup consists of nine Ascomycete phytochromes from *Sclerotinia sclerotiorum*, *Botryotinia fuckeliana*, *Neurospora crassa* and *Phaeosphaeria nodorum* (Additional file 1). A few of these phytochromes are also represented in the basal fungal subgroup in HK trees. These species exhibit at least one other phytochrome placed at a more advanced position. Thus, the last common ancestor of Ascomycetes and Basidiomycetes exhibited two phytochromes, one of which was lost in Basidiomycetes and in some Ascomycetes but retained in other Ascomycetes, such as *Neurospora crassa* (Figure 6). The evolution of phytochromes in fungi is comparable in this regard with that in streptophytes, as both lineages involved a gene duplication event that must have occurred during the early phase of that particular group.

**Table 8:** **Basidiomycete phytochromes appear as a subgroup of Ascomycete phytochromes**

	**PGP trees (total of 16) number of trees**	**HK trees (total of 11) number of trees**
Basidiomycetes as one of five fungal subgroups	15	6
Basidiomycetes sister of Ascomycetes	0	4

## Conclusions

Phylogenetic studies that cover a long time range are usually hampered by unclear branch positions. Bootstrapping indicates the reliability of branches but does not provide an alternative if a value is low. Here, we use multiple programs for alignment and tree construction. Manual inspection of these various trees with respect to specific questions allowed us to reach reliable conclusions regarding the major steps in the evolution of pro- and eukaryotic phytochromes.

Since manual interpretation is required, the present method does still have limitations. Also, the choice of programs and replacement matrices could still affect the outcome. We nevertheless assume that the “multiple tree approach” is superior over studies where only few calculations are performed, especially in situations where branching is insecure.

It was found that horizontal gene transfer of phytochrome genes has occurred frequently between various bacterial groups and that the C-terminal HK has often been replaced by another HK. This type of event underlies the evolution of plant phytochromes with an N-terminus of cyanobacterial origin and a C-terminus that originated from other prokaryotes. The evolutionary advantage of this rearrangement could be that nuclear genes are brought under the control of a phytochrome sensor that is adapted to the spectral characteristics of chlorophyll. Without this rearrangement, phytochrome would be specialized to control genes of the endosymbiont, i.e., plastidic genes, or would exhibit a BV chromophore. The gene duplication event during the early evolution of two lineages, streptophytes and fungi, suggests a general principle in the early evolution of eukaryotes, which could be coupled to genome duplication.

## Methods

Phytochrome protein sequences were retrieved from the GenBank database after extensive NCBI BLAST searches employing five plant, three bacterial, two fungal and five cyanobacterial phytochrome sequences as queries. Using the domain prediction program PFAM (http://pfam.sanger.ac.uk/), sequences with PAS, GAF, PHY domains and HK sequences were selected. After removing duplicates, a collection of 442 protein sequences was obtained, which was used for alignments and tree constructions. This collection included 246 bacterial phytochromes, 56 fungal phytochromes, 137 land plant phytochromes, two phytochromes from green algae (*Mougeotia scalaris* and *Mesotaenium caldariorum*, Zygnematophyceae) and one phytochrome from the diatom *Thalassiosira pseudonana*. Prasinophyte or glaucophyte phytochrome sequences were not included in our analyses because they were added to the databases after we had initiated alignment and tree construction. The bacterial collection encompassed 29 sequences from cyanobacteria, 92 from α proteobacteria, 20 from ß proteobacteria, 62 from γ proteobacteria, 19 from Bacteroidetes and 24 from other taxa. The sequence IDs, species names and phylogenetic groups are provided in Additional file 3. From each sequence, the N-terminal PGP and the C-terminal HK were extracted; other sequence information was discarded. The PGP and HK sequences were aligned using the programs MUSCLE [68] and MAFFT version 6.81 [69], resulting in four sets of aligned sequences. The MAFFT parameters were as follows: BLOSUM62 scoring matrix, gap open penalty 1.53, offset value 0.123 and FFT-NS-2 strategy. With MUSCLE, default parameters were used. The alignments were edited with JALVIEW (http://www.jalview.org) by deleting positions with more than 50% gaps. From this procedure, the frequency of phylogenetically informative sites in the PGP region was increased from 80% to 99.5%, as indicated by the Topali program [70]. For the HK region, the value increased from 97% to 100%. Six programs were used for tree construction: FITCH; NeigHbor; Protpars; and ProML from the program package Phylip version 3.69 [71], PyML version 3.1 [72] and MrBayes version 3.2.2 [73]. In the PHYLIP programs, the default parameters were used: JTT distance matrix [74], no “gamma distribution of rates among positions” and no “outgroup root.” Most programs were also combined with bootstrapping. In those cases, 100 datasets were generated, and consense trees were calculated according to the majority rule. The branch lengths of the consense results were estimated using ProML with the consensus trees as user trees. Prior to the construction of HK trees with NEIGHBOR, FITCH or PROTPARS, 33 sequences were deleted because their overlaps were too short. With PhyML, the default value of 4 for the number of substitution rate categories was always selected. With this program, seven different replacement matrices were used. For each matrix, the log likelihood for parameter combinations (+I, estimated proportion of invariable sites; +G, estimated gamma distribution parameter; +F, empirical amino acid frequencies) was estimated with prottest3 [75]. The parameter combinations for the best-fit models were selected. The estimated running time for MrBayes with the large datasets was too long (estimated at several months). For this reason, the number of sequences was reduced to 152. For this reduction, information from the other trees was considered: in each cluster that was found in most trees, all of the sequences except one were deleted. MrBayes was run using a fixed WAG model, with 4 parallel runs and approximately 5,000,000 generations until the deviation of split frequencies fell below 0.5. Circular tree graphics were prepared with ITOL (http://itol.embl.de) employing different colors for each of the major groups. Each sequence is denominated by a 5-letter code that stands for the species (the first three letters of the first and the first two letters for the second part of the species name). If distinct strains belonging to the same species are present, the code of the second strain contains an additional letter or digit. The name of the protein is abbreviated as Phy followed by a letter for streptophytes; Fph followed by a letter or number for fungal phytochromes; CphA or CphB for cyanobacterial phytochromes; and BphP for bacterial phytochromes. Examples of PGP and HK trees are shown in Figures 2 and 3; Additional files 1 and 2 show all trees.

## Additional files

Additional file 1:
**Phylogenetic PGP trees P1 to P17.** Parameter settings are given in Additional file 4. Additional file 2:
**Phylogenetic HK trees H1 to H13.** Parameter settings are given in Additional file 5. Additional file 3:
**List of phytochrome sequences, abbreviations, access numbers, phylogenetic grouping.**
Additional file 4:
**Parameter settings and results of PGP trees.** The trees are shown in Additional file 2. Column 4 indicates the used replacement matrix (JTT, Jones Taylor Thornton [76], WAG, Wheelan and Goldman [77], LG, Le Gascuel [78]) and non-default parameter settings: “I”, estimated proportion of invariable sites, “G”, estimated gamma distribution parameter,“F”, empirical amino acid frequencies bacteria. Additional file 5:
**Parameter settings and results of HK trees.** The trees are shown in Additional file 2. Column 4 indicates the used replacement matrix (JTT, Jones Taylor Thornton [76], WAG, Wheelan and Goldman [77], LG, Le Gascuel [78]) and non-default parameter settings: “I”, estimated proportion of invariable sites, “G”, estimated gamma distribution parameter,“F”, empirical amino acid frequencies. Additional file 6:
**Phylogenetic trees constructed with PAS/PAS/HK of two streptophyte phytochromes and prokaryotic proteins.**

## Abbreviations

BV: Biliverdin
HK: Histidine kinase (like) region
PGP: Region containing an N-terminal PAS, central GAF and C-terminal PHY domain
PAS domain: Protein domain denominated according to the founding members PER ARNDT SIM
GAF domain: Protein domain abbreviated according to the founding members c**G**MP-specific phosphodiesterases, **a**denylyl cyclases and **F**hlA [25]
PCB: Phycocyanobilin
PϕB: Phytochromobilin
