# Supplementary material for: Streptophyte phytochromes exhibit an N-terminus of cyanobacterial origin and a C-terminus of proteobacterial origin
Source: BMC Res Notes. 2015 Apr 13;8:144. doi: 10.1186/s13104-015-1082-3 (PMC4422448; doi:10.1186/s13104-015-1082-3)
Supplement: Additional file 2: — Phylogenetic HK trees H1 to H13. Parameter settings are given in Additional file 5. [file 13104_2015_1082_MOESM2_ESM.pdf]

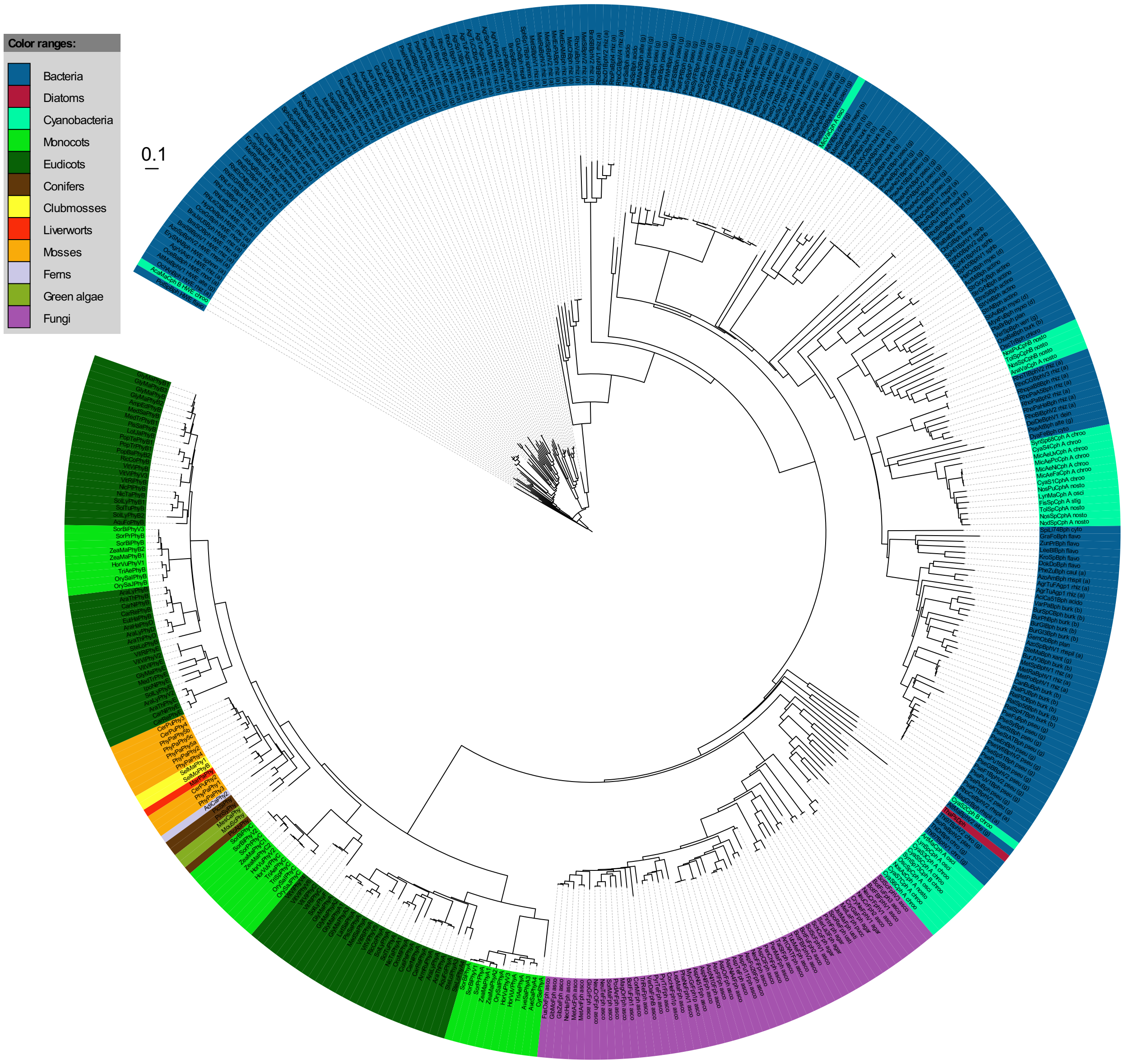
Color ranges:
Bacteria
Diatoms
Cyanobacteria
Monocots
Eudicots
Conifers
Clubmosses
Liverworts
Mosses
Ferns
Green algae
Fungi
0.1

H1

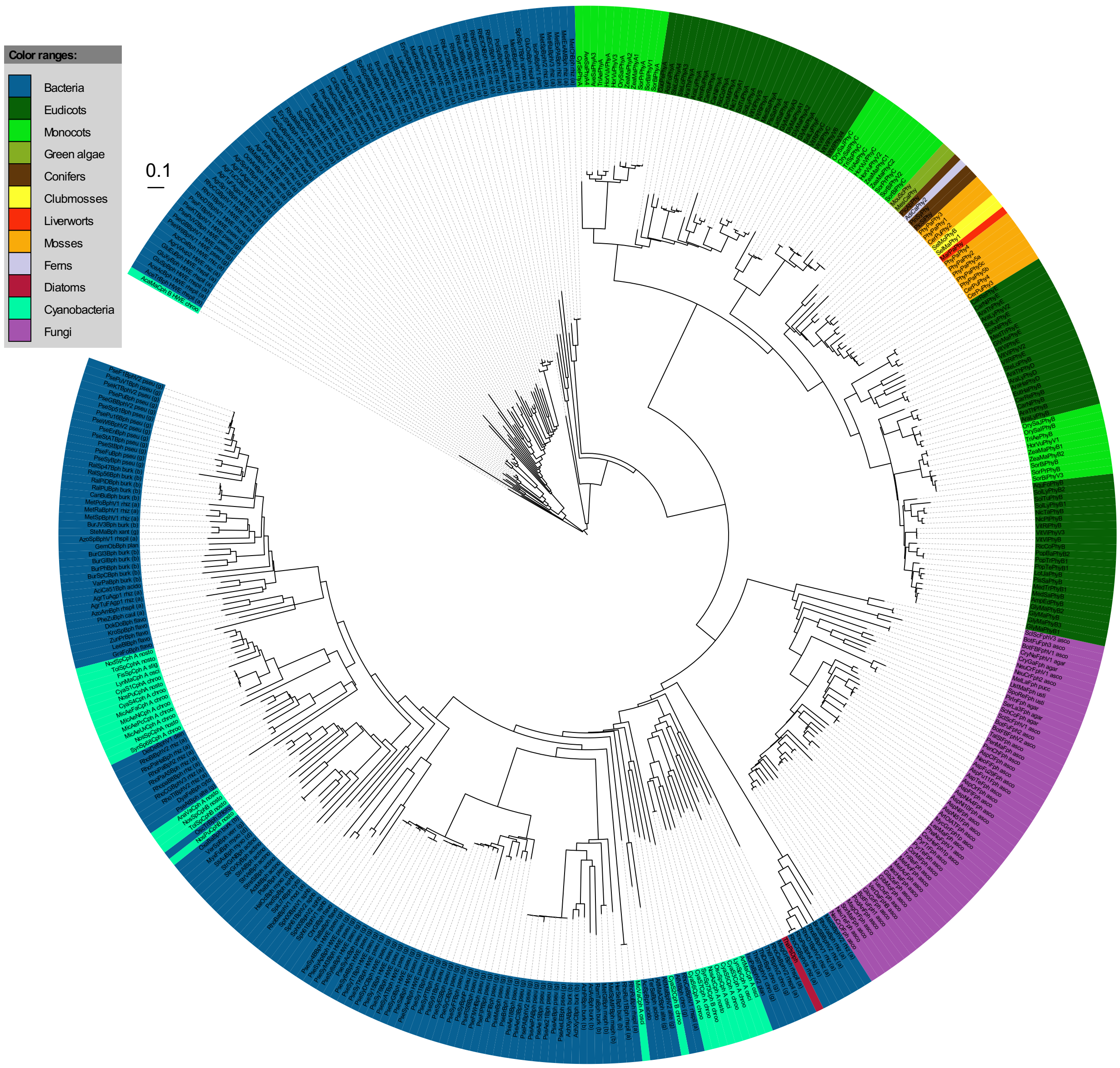
Color ranges:
Bacteria
Eudicots
Monocots
Green algae
Conifers
Clubmosses
Liverworts
Mosses
Ferns
Diatoms
Cyanobacteria
Fungi
0.1

H2

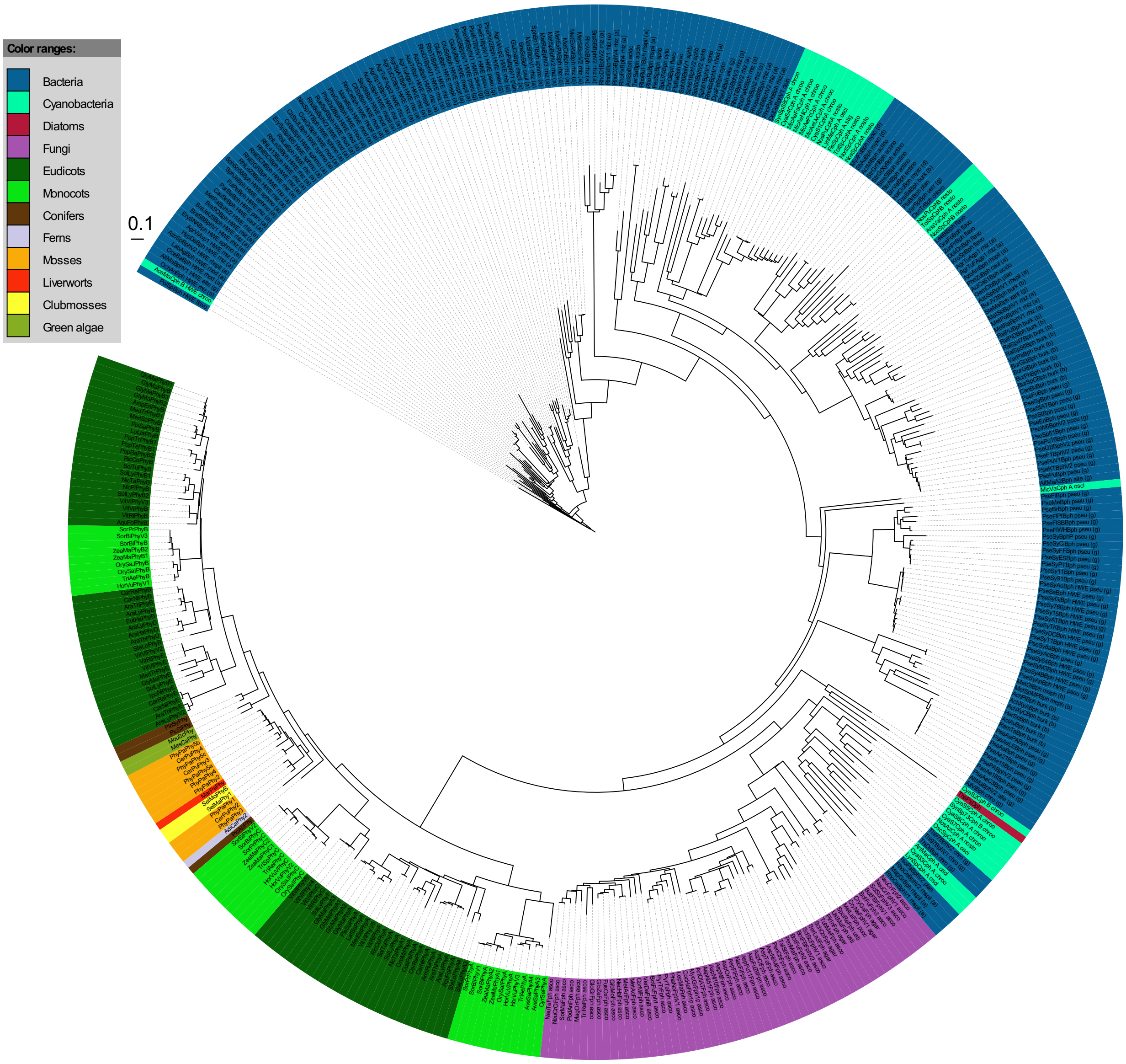
Color ranges:
Bacteria
Cyanobacteria
Diatoms
Fungi
Eudicots
Monocots
Conifers
Ferns
Mosses
Liverworts
Clubmosses
Green algae
0.1

H3

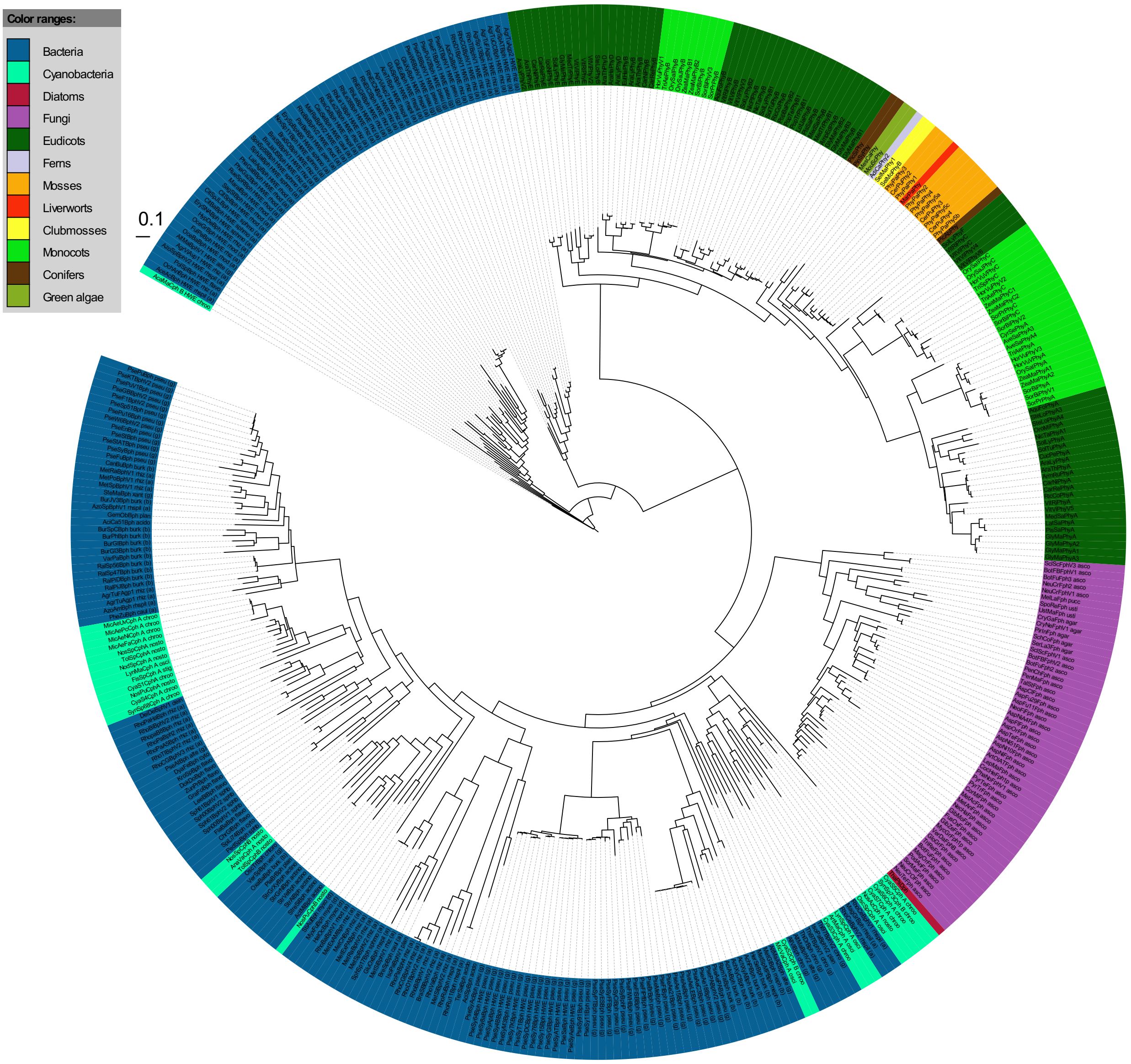
Color ranges:
Bacteria
Cyanobacteria
Diatoms
Fungi
Eudicots
Ferns
Mosses
Liverworts
Clubmosses
Monocots
Conifers
Green algae
0.1

H4

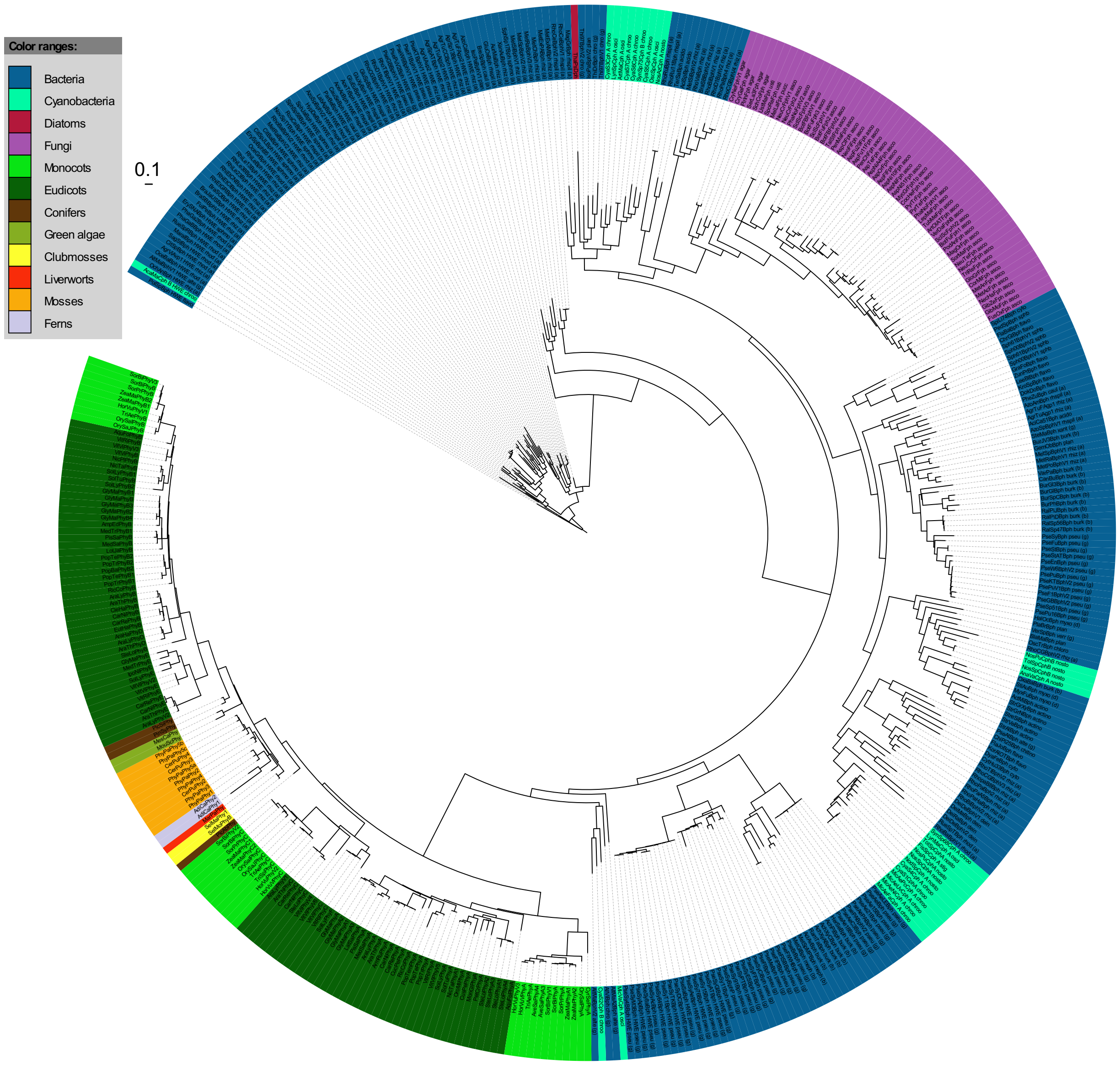
Color ranges:
Bacteria
Cyanobacteria
Diatoms
Fungi
Monocots
Eudicots
Conifers
Green algae
Clubmosses
Liverworts
Mosses
Ferns
0.1

H5

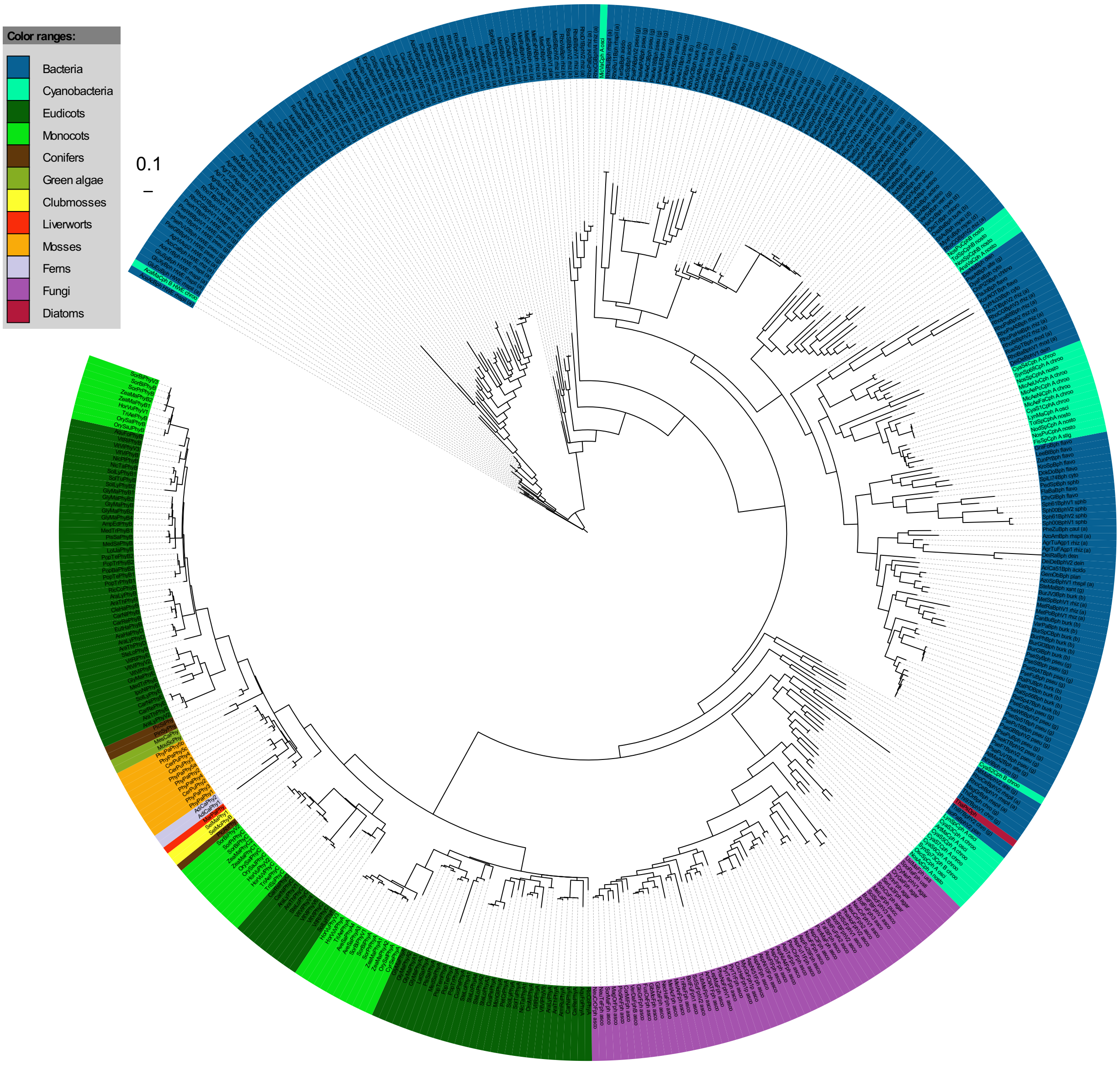
Color ranges:
Bacteria
Cyanobacteria
Eudicots
Monocots
Conifers
Green algae
Clubmosses
Liverworts
Mosses
Ferns
Fungi
Diatoms
0.1

H6

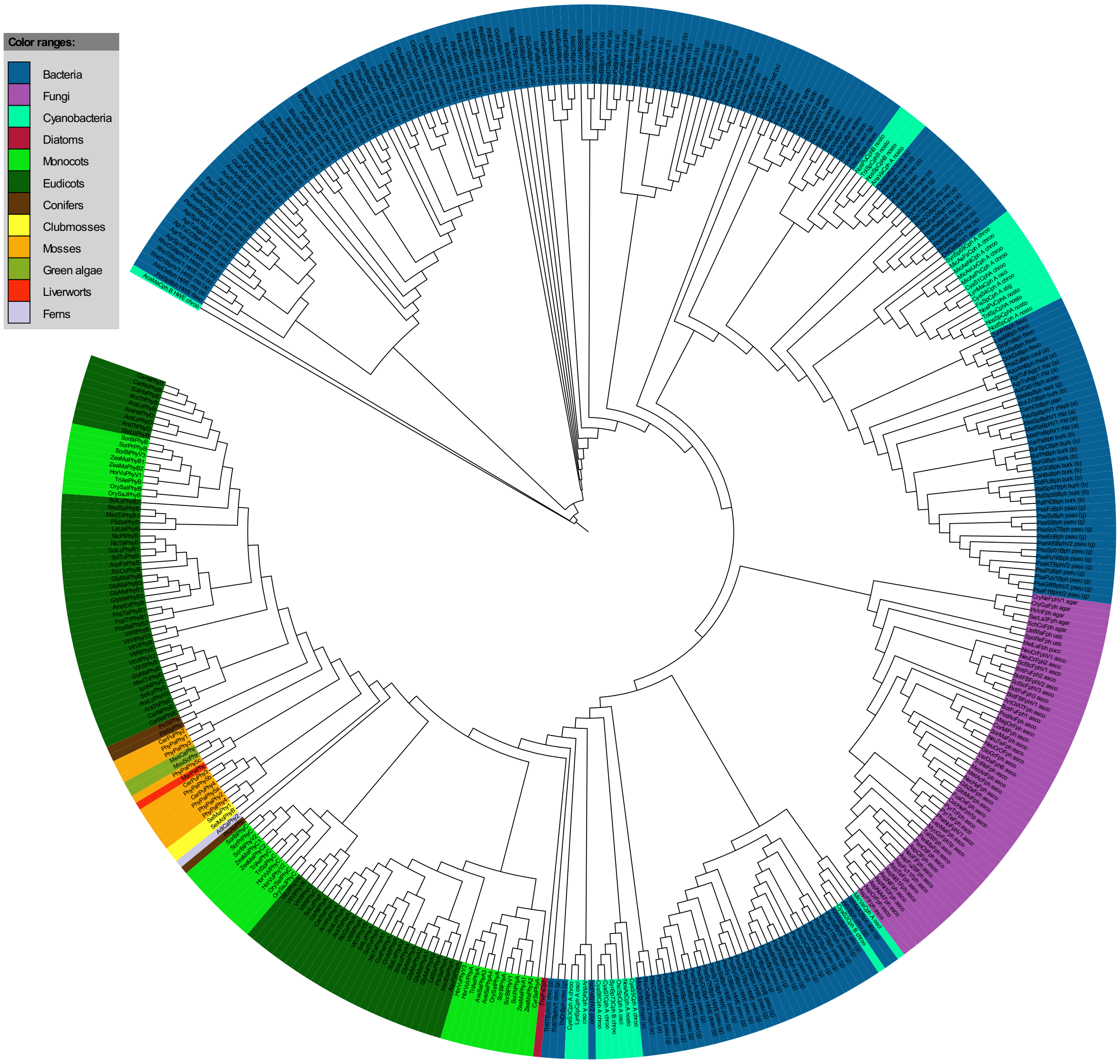
Color ranges:
Bacteria
Fungi
Cyanobacteria
Diatoms
Monocots
Eudicots
Conifers
Clubmosses
Mosses
Green algae
Liverworts
Ferns

H7

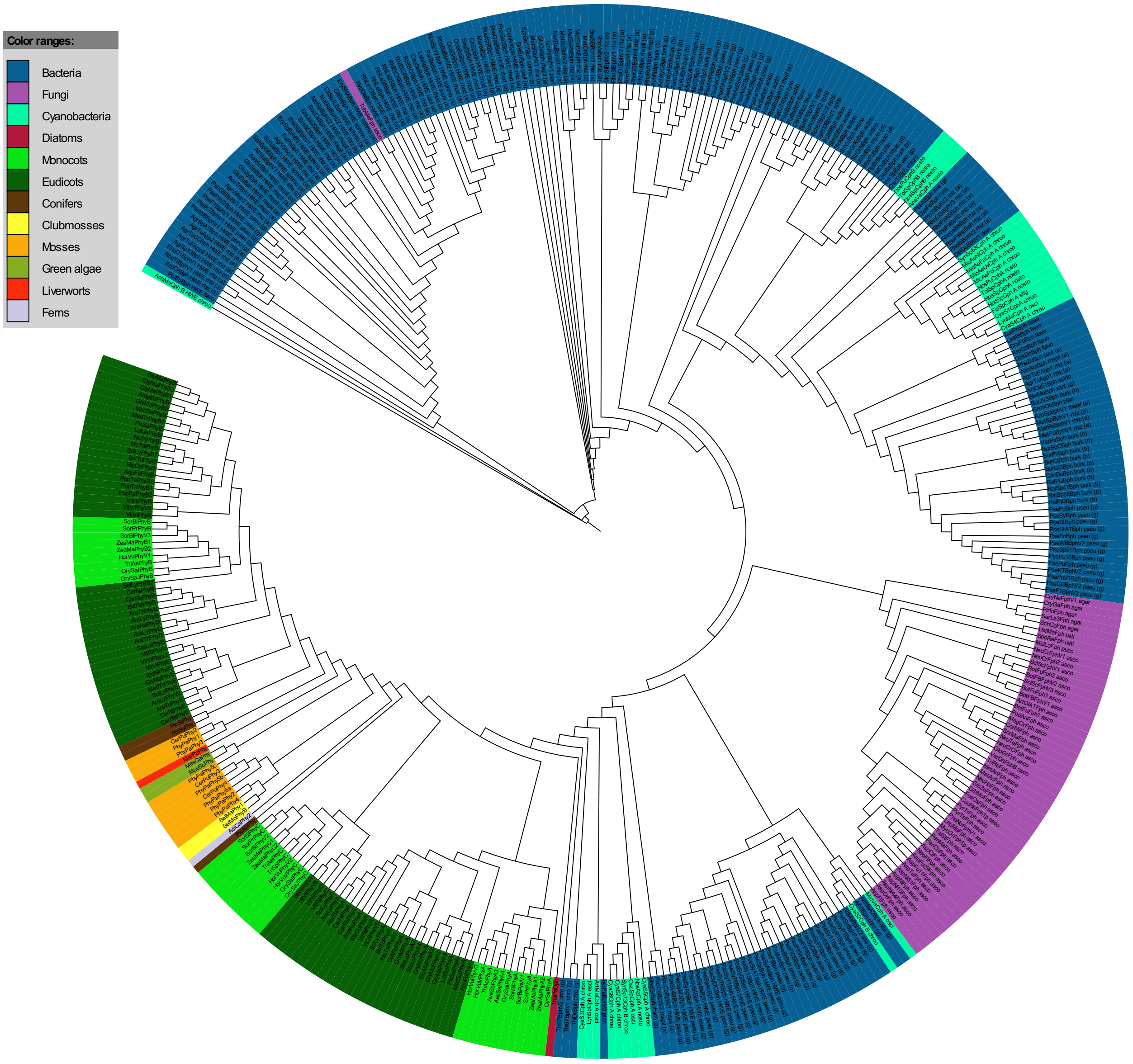
Color ranges:
Bacteria
Fungi
Cyanobacteria
Diatoms
Monocots
Eudicots
Conifers
Clubmosses
Mosses
Green algae
Liverworts
Ferns

H8

H9

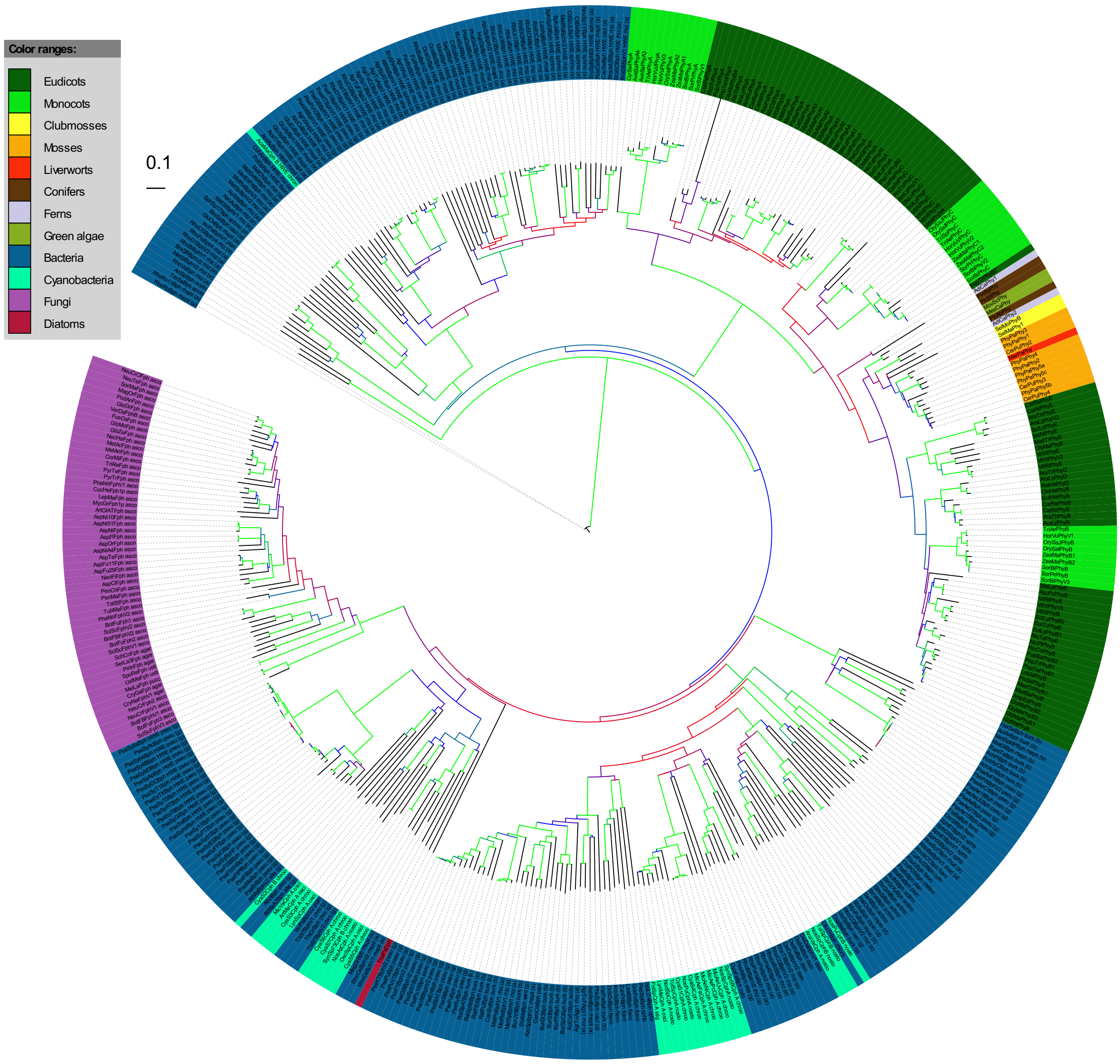
Color ranges:
Eudicots
Monocots
Clubmosses
Mosses
Liverworts
Conifers
Ferns
Green algae
Bacteria
Cyanobacteria
Fungi
Diatoms
0.1

H10

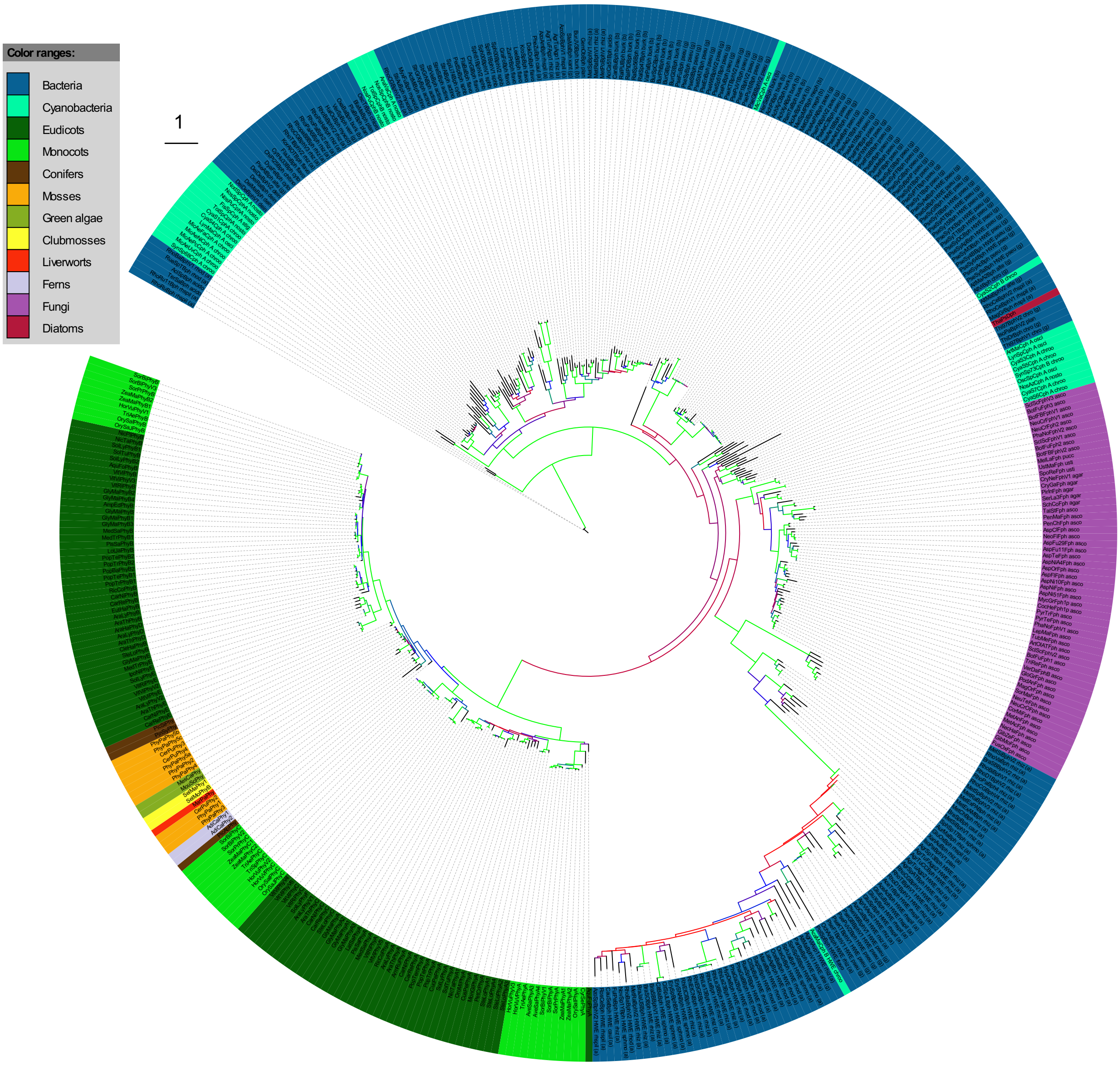
Color ranges:
Bacteria
Cyanobacteria
Eudicots
Monocots
Conifers
Mosses
Green algae
Clubmosses
Liverworts
Ferns
Fungi
Diatoms
1

H11

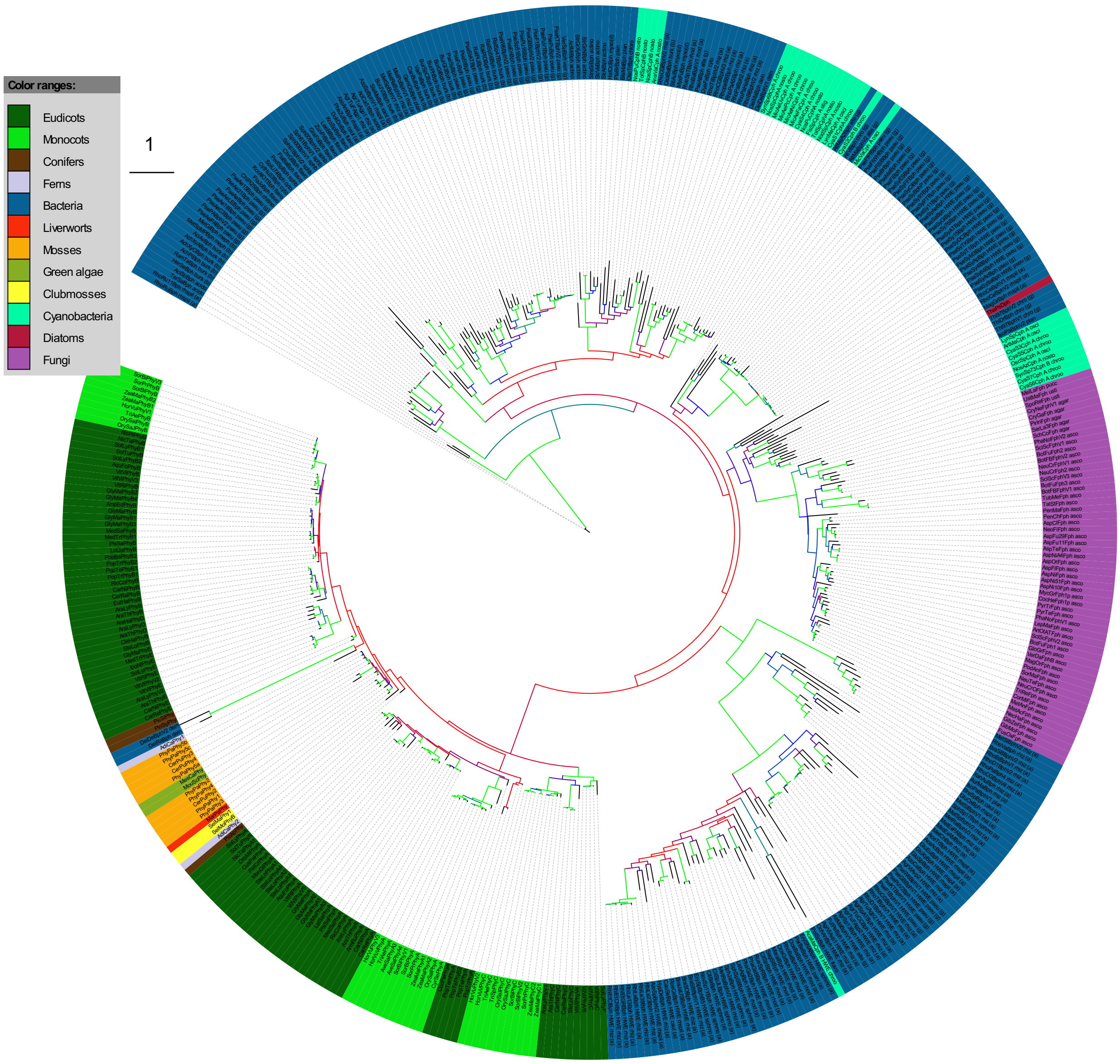
Color ranges:
Eudicots
Monocots
Conifers
Ferns
Bacteria
Liverworts
Mosses
Green algae
Clubmosses
Cyanobacteria
Diatoms
Fungi
1

H12

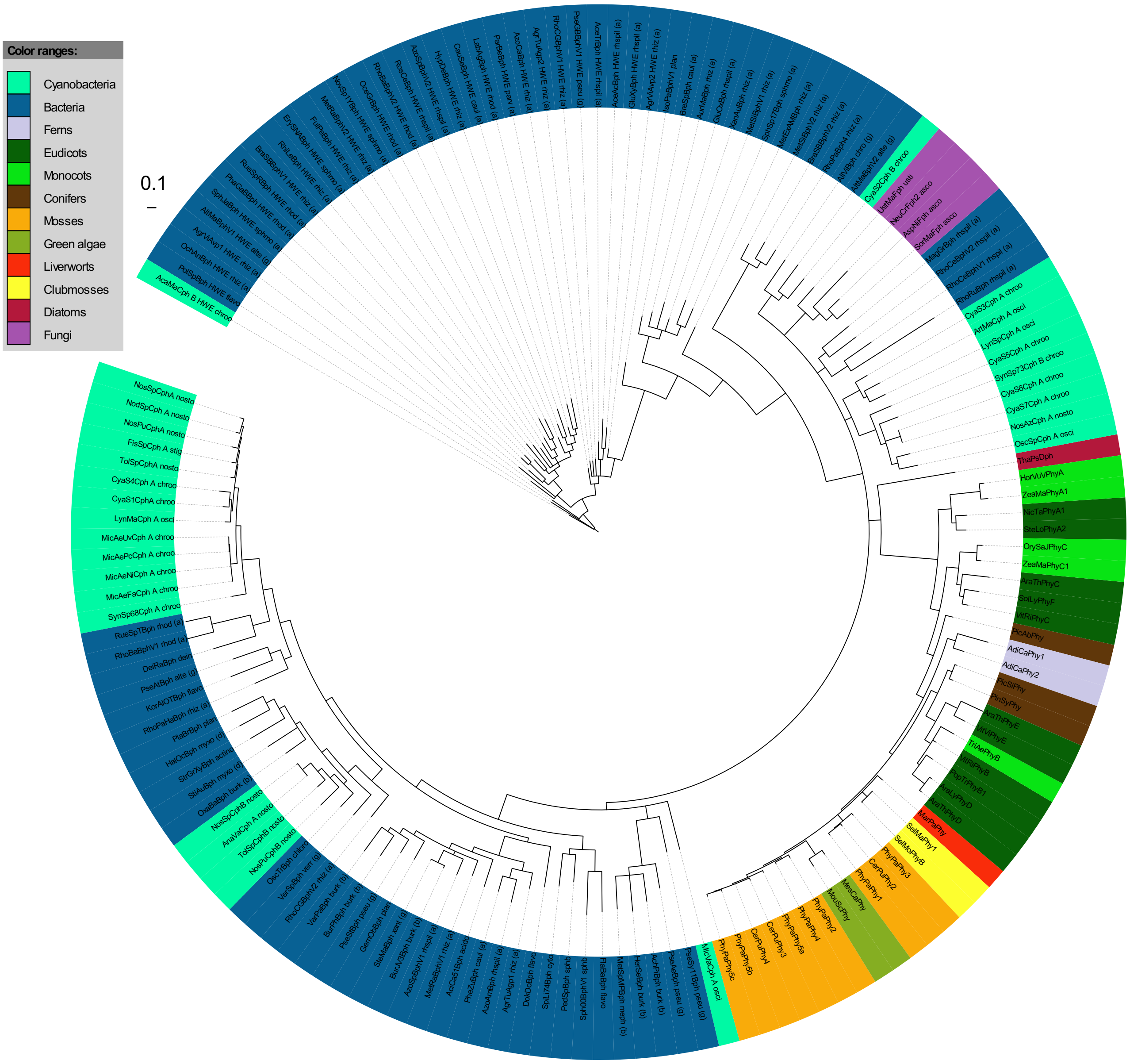

H13

Additional file 3. Phylogenetic HK trees H1 to H13. The tree numbers refer to Table 2 which gives details for tree construction and a summary. The color code is: blue for bacteria, light blue for cyanobacteria, orange for mosses, green for dicotyledonous plants, light green for monocotyledonous plants, brown for gymnosperms, red for liverworts, grey for ferns, purple for fungi and dark violet for diatom. Typically, phytochromes are denominated by a 5 letters species code together with an abbreviation for the particular group of phytochrome. Accession numbers and species / strain names are listed in supplemental Table 1. After each phytochrome abbreviation, additional taxonomic information is given for bacterial and fungal sequences: (a), α- proteobacterium; (b), ß-proteobacterium; (g), γ-proteobacterium; (d), δ-proteobacterium; acido, Adicobacteriales; actino, Actinomycetales; agar, Agaricomycetes (basidiomycetes); asco, Ascomycota; burk, Burkholderiales; caul, Caulobacterales; chitino, Chitinophagaceae; chroo, Chroococcales; cyto, Cytophagales; dein, Deinococcales; flavo, Flavobacteria; HWE, HWE histidine kinase; meph, Methylophilales; myxo, Myxococcales; nosto, Nostocales; osci, Oscillatoriales; parv, Parvulaculales; plan, Planctomycetales; pseu, Pseudomonadales; pucci, Pucciniomycotes (basidiomycetes); rhizo, Rhizobiales; rhod, Rhodobacterales; rhspil, Rhodospirillales; sphmo, Sphingomonadales; stig, Stigonematales; usti, Ustilagomycotina (basidiomycetes); verr, Verrucomicrobiales; xant, Xanthomonadales. The color of the branches indicate bootstrap values of 10-30% (red), 80-100% (green). Intermediate values are colored blue or with mixed colors.
