# Supplementary material for: Streptophyte phytochromes exhibit an N-terminus of cyanobacterial origin and a C-terminus of proteobacterial origin
Source: BMC Res Notes. 2015 Apr 13;8:144. doi: 10.1186/s13104-015-1082-3 (PMC4422448; doi:10.1186/s13104-015-1082-3)
Supplement: Additional file 4: — Parameter settings and results of PGP trees. The trees are shown in Additional file 2. Column 4 indicates the used replacement matrix (JTT, Jones Taylor Thornton [76], WAG, Wheelan and Goldman [77], LG, Le Gascuel [78]) and non-default parameter settings: “I”, estimated proportion of invariable sites, “G”, estimated gamma distribution parameter,“F”, empirical amino acid frequencies bacteria. [file 13104_2015_1082_MOESM4_ESM.pdf]

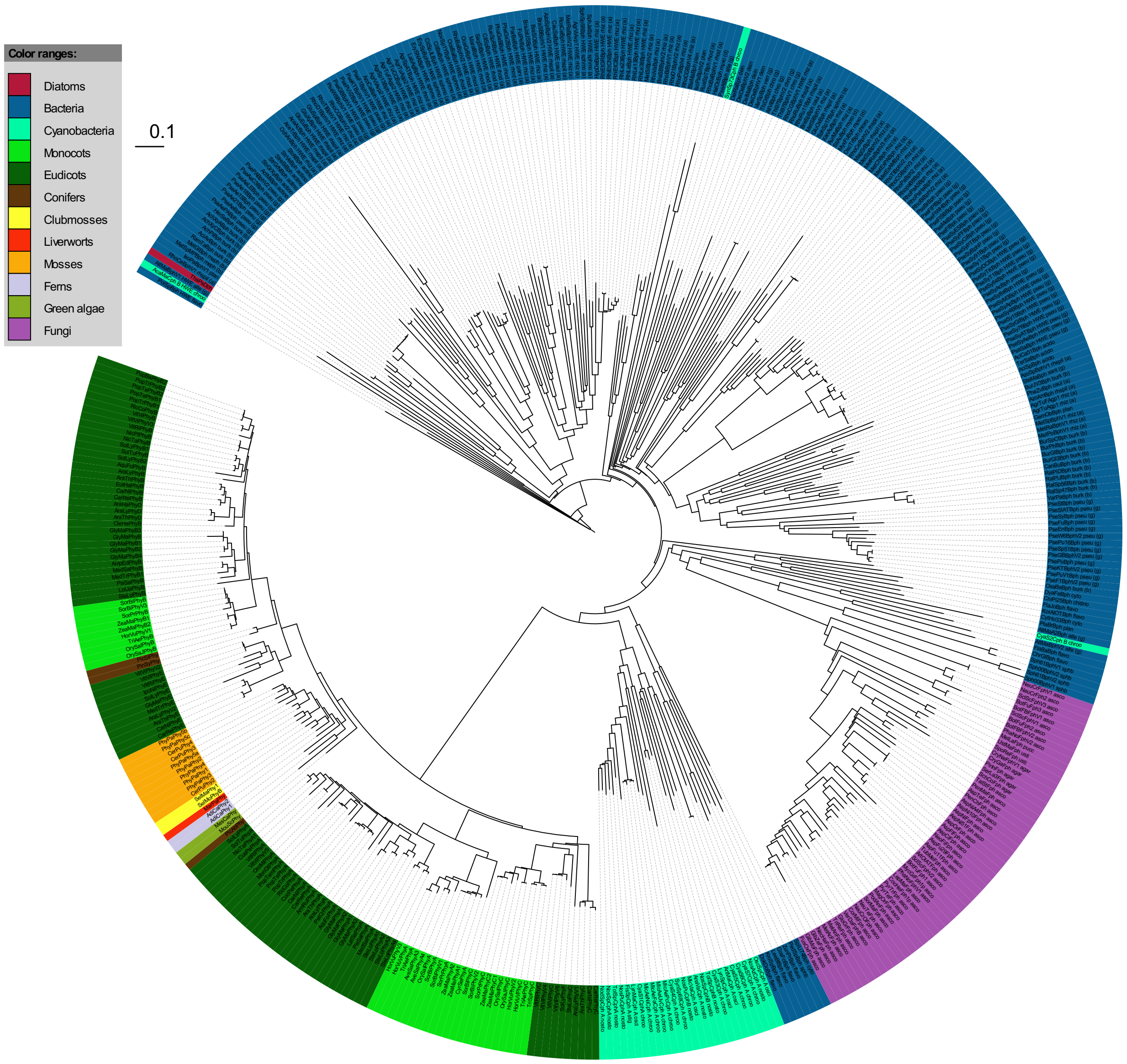
Color ranges:
Diatoms
Bacteria
Cyanobacteria
Monocots
Eudicots
Conifers
Clubmosses
Liverworts
Mosses
Ferns
Green algae
Fungi
0.1

P1

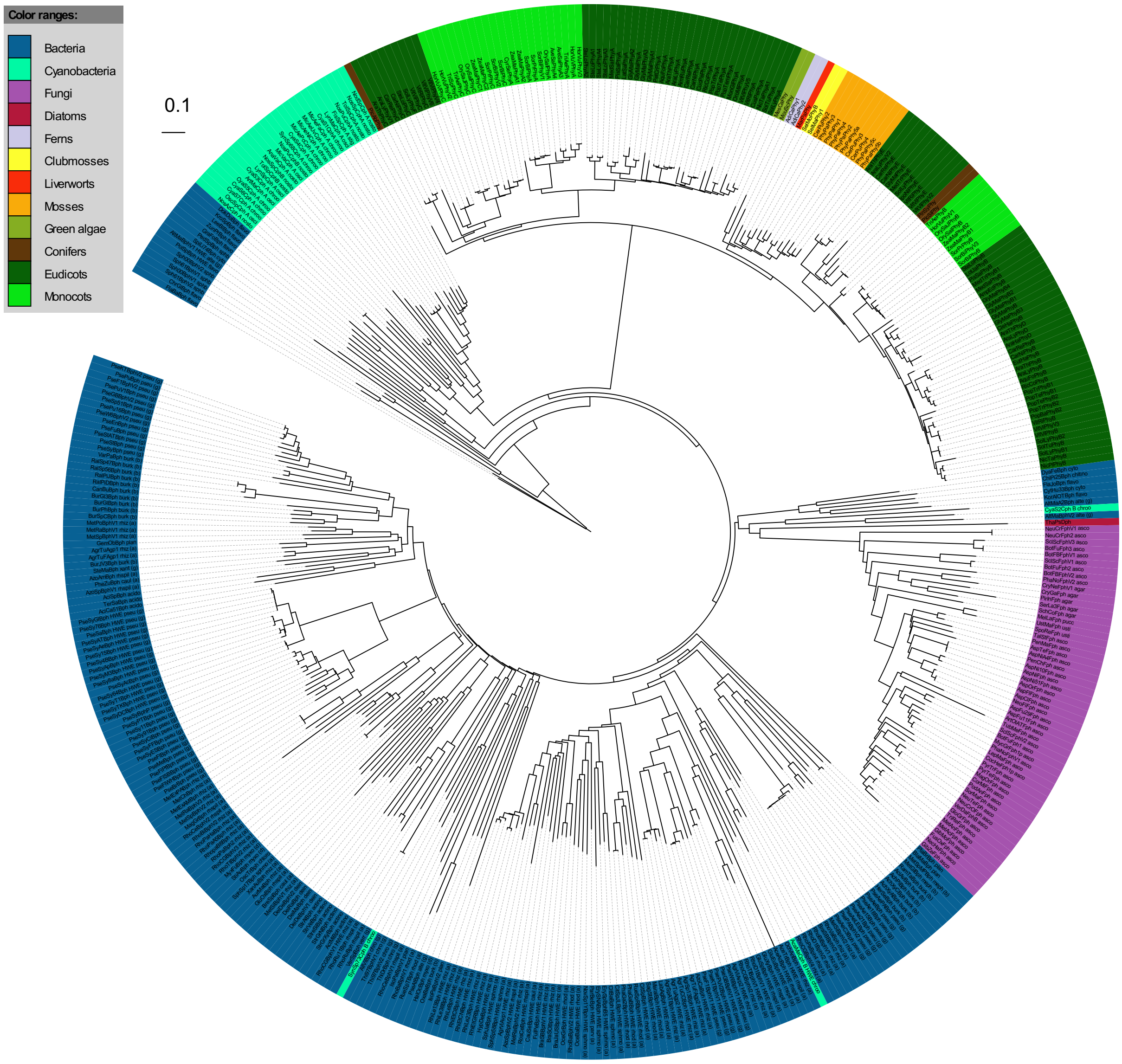
Color ranges:
Bacteria
Cyanobacteria
Fungi
Diatoms
Ferns
Clubmosses
Liverworts
Mosses
Green algae
Conifers
Eudicots
Monocots
0.1

P2

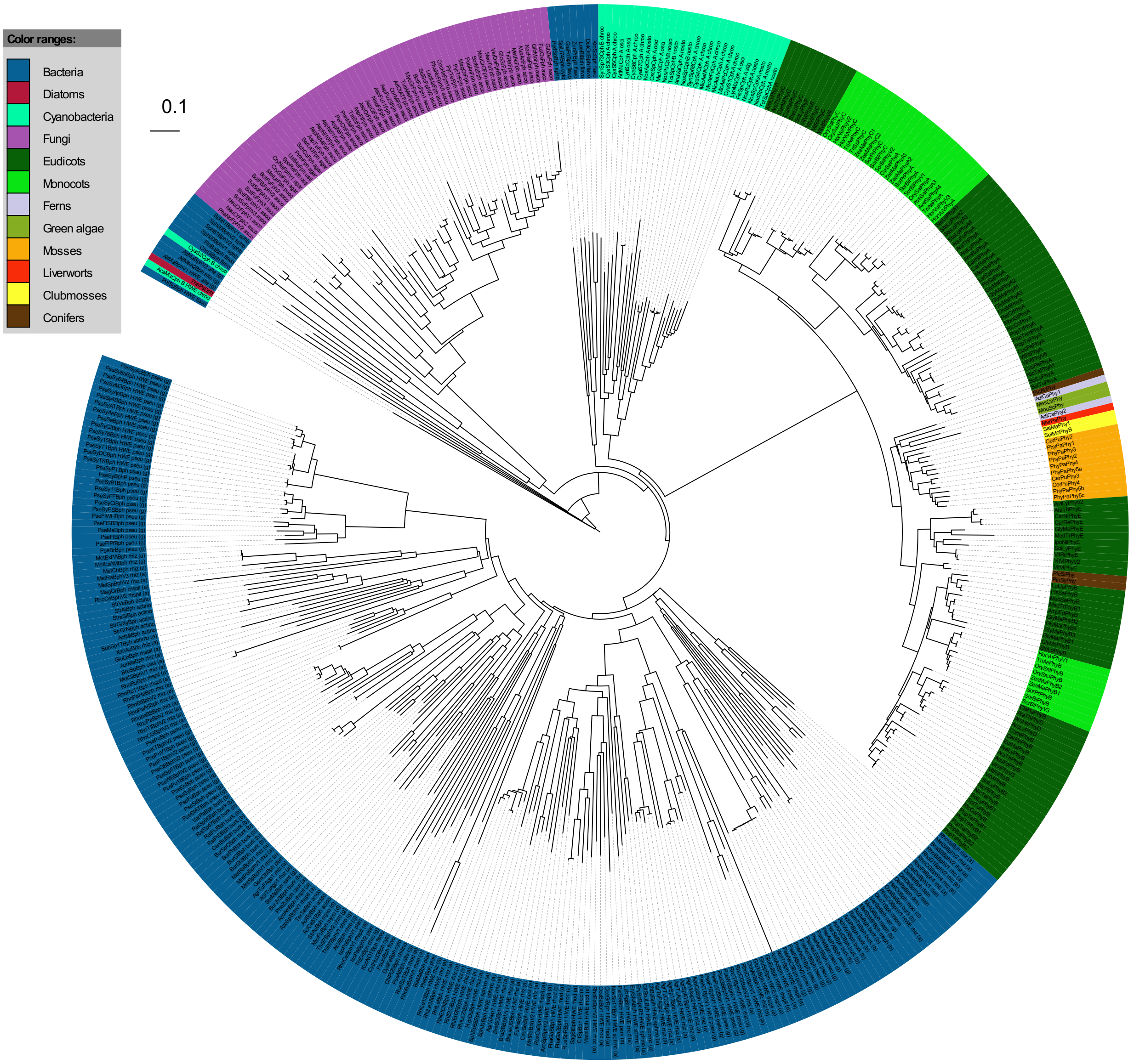
Color ranges:
Bacteria
Diatoms
Cyanobacteria
Fungi
Eudicots
Monocots
Ferns
Green algae
Mosses
Liverworts
Clubmosses
Conifers
0.1

P3

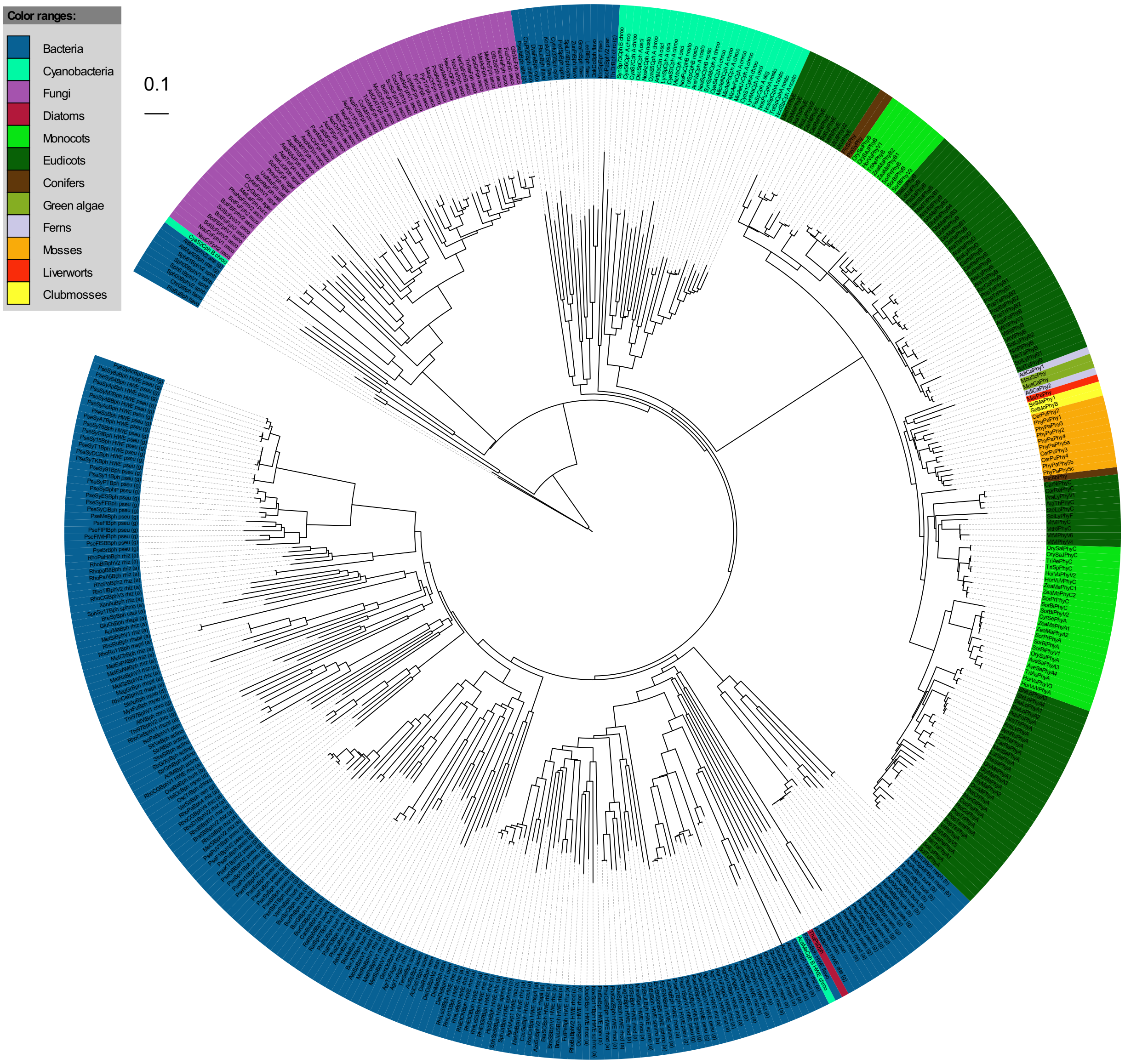

Color ranges:
Bacteria
Cyanobacteria
Fungi
Diatoms
Monocots
Eudicots
Conifers
Green algae
Ferns
Mosses
Liverworts
Clubmosses
0.1

P4

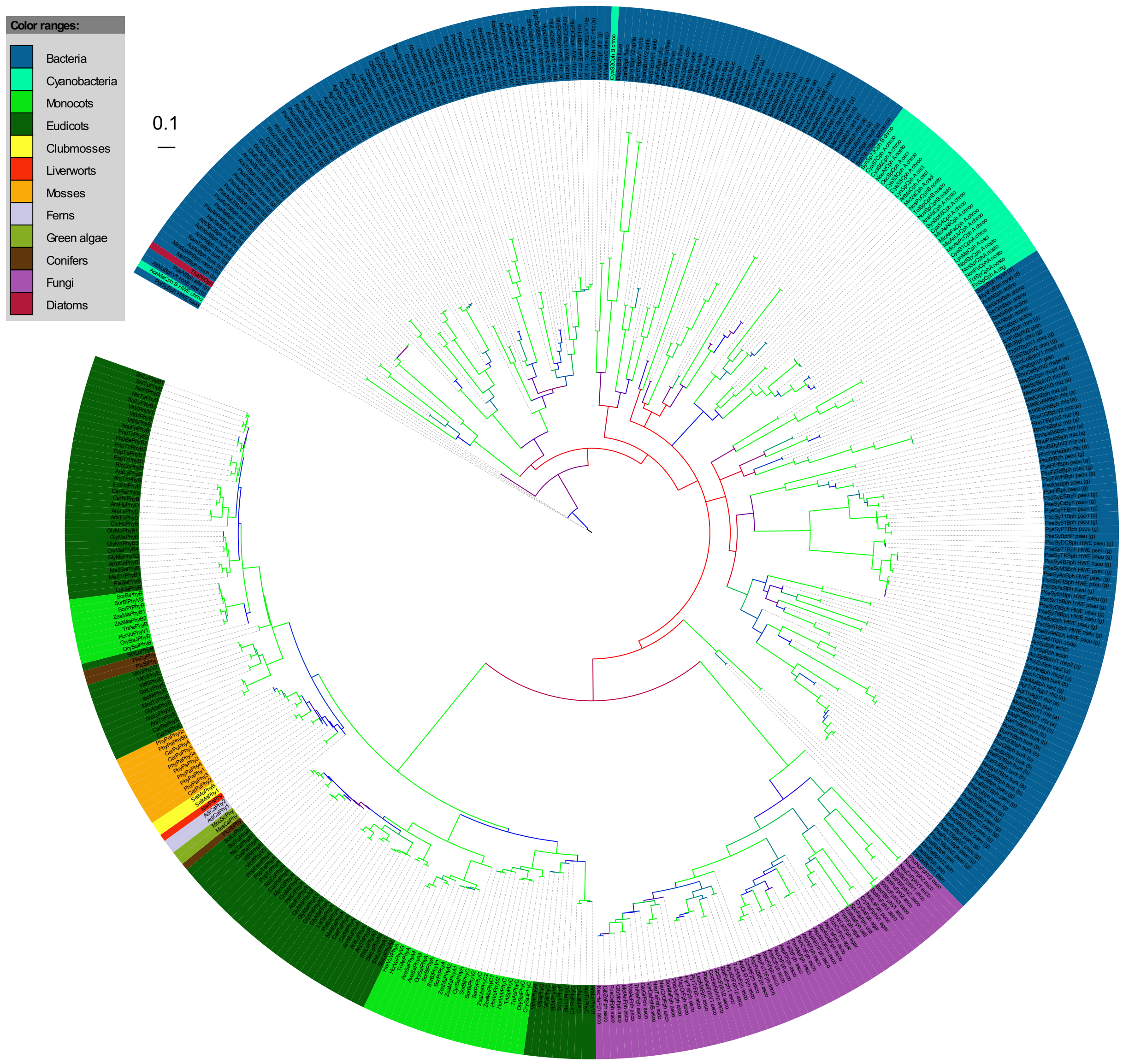
Color ranges:
Bacteria
Cyanobacteria
Monocots
Eudicots
Clubmosses
Liverworts
Mosses
Ferns
Green algae
Conifers
Fungi
Diatoms
0.1

P5

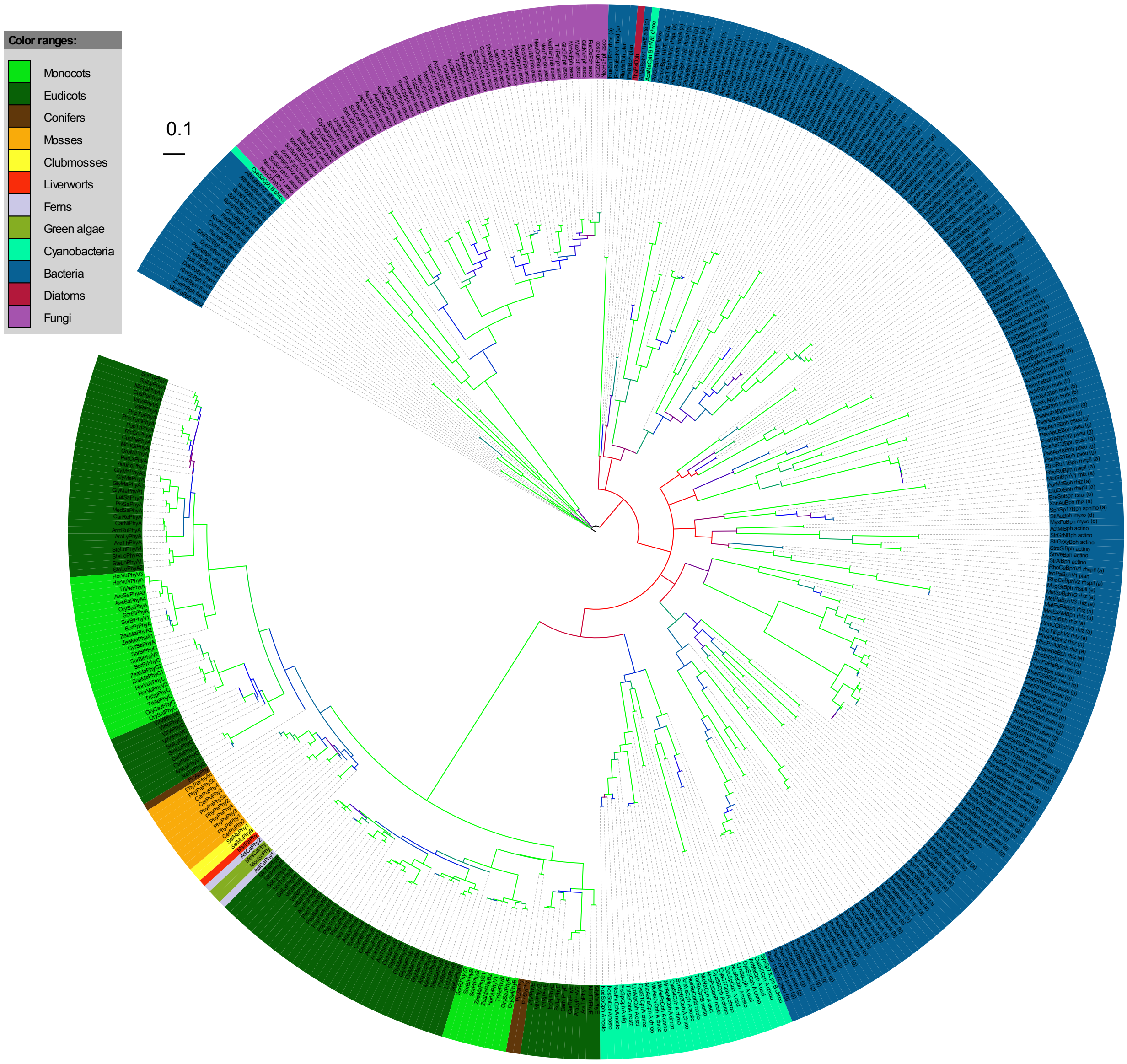
Color ranges:
Monocots
Eudicots
Conifers
Mosses
Clubmosses
Liverworts
Ferns
Green algae
Cyanobacteria
Bacteria
Diatoms
Fungi
0.1

P6

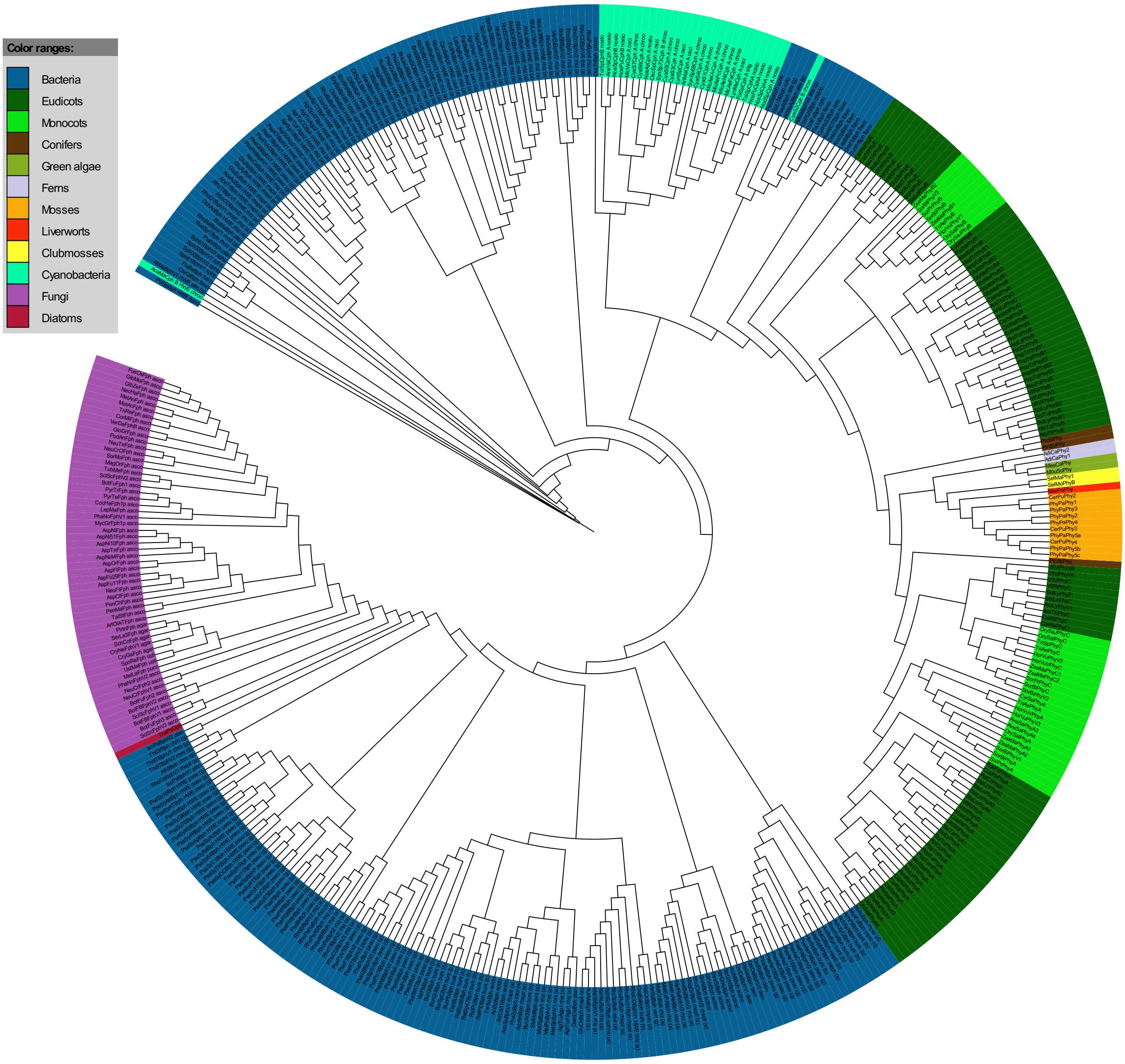
Color ranges:
Bacteria
Eudicots
Monocots
Conifers
Green algae
Ferns
Mosses
Liverworts
Clubmosses
Cyanobacteria
Fungi
Diatoms

P7

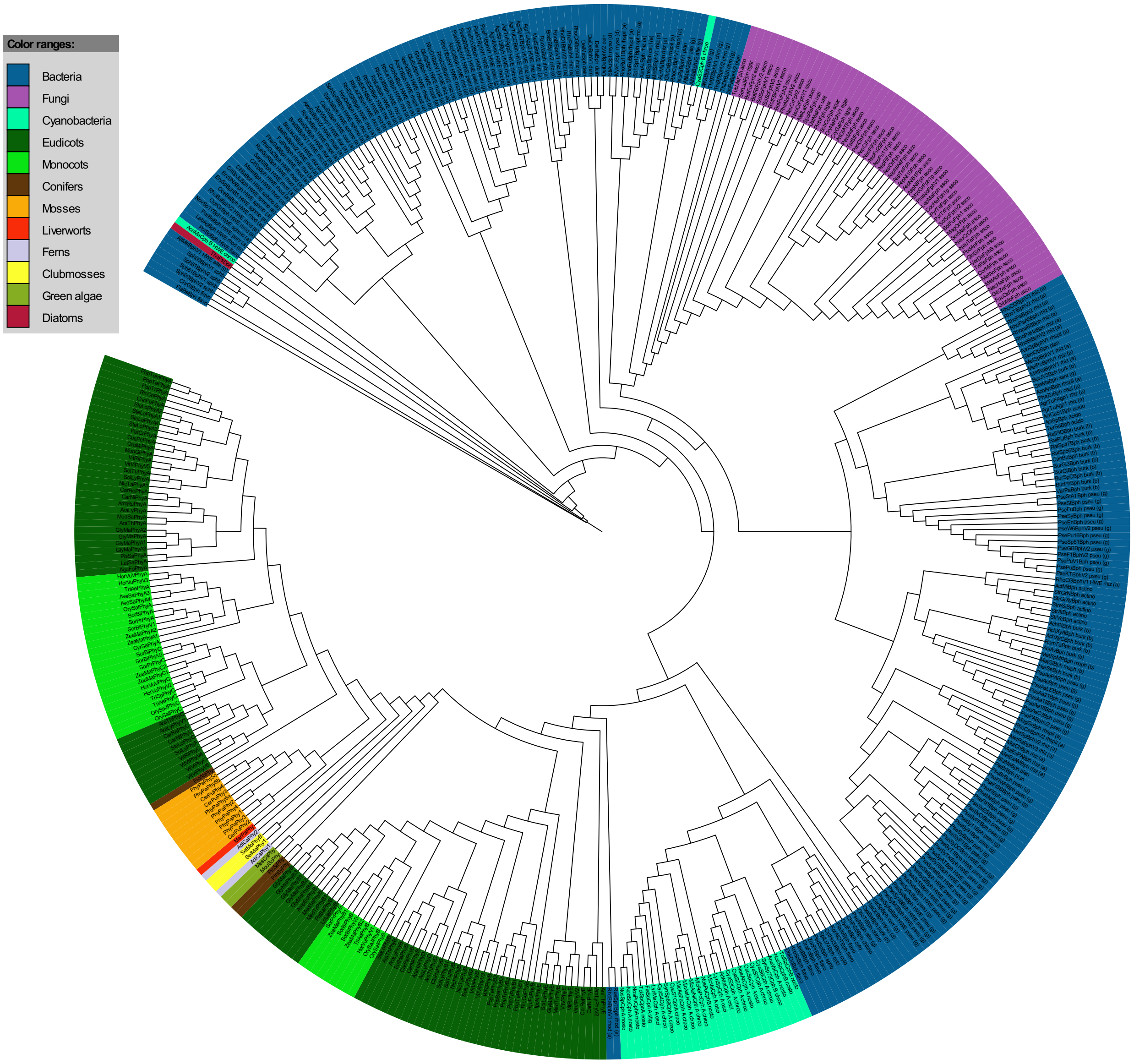
Color ranges:
Bacteria
Fungi
Cyanobacteria
Eudicots
Monocots
Conifers
Mosses
Liverworts
Ferns
Clubmosses
Green algae
Diatoms

P8

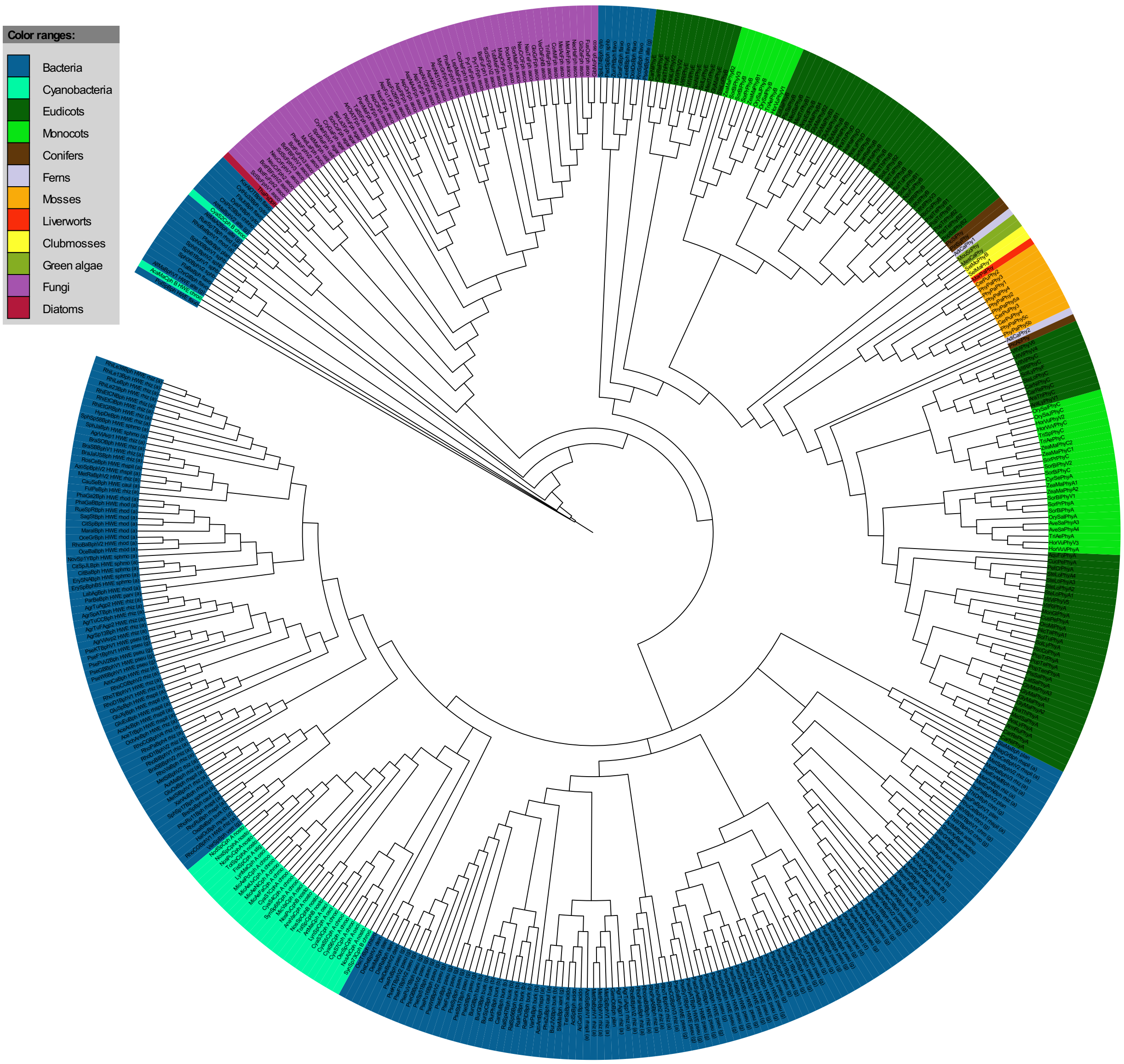
Color ranges:
Bacteria
Cyanobacteria
Eudicots
Monocots
Conifers
Ferns
Mosses
Liverworts
Clubmosses
Green algae
Fungi
Diatoms

P9

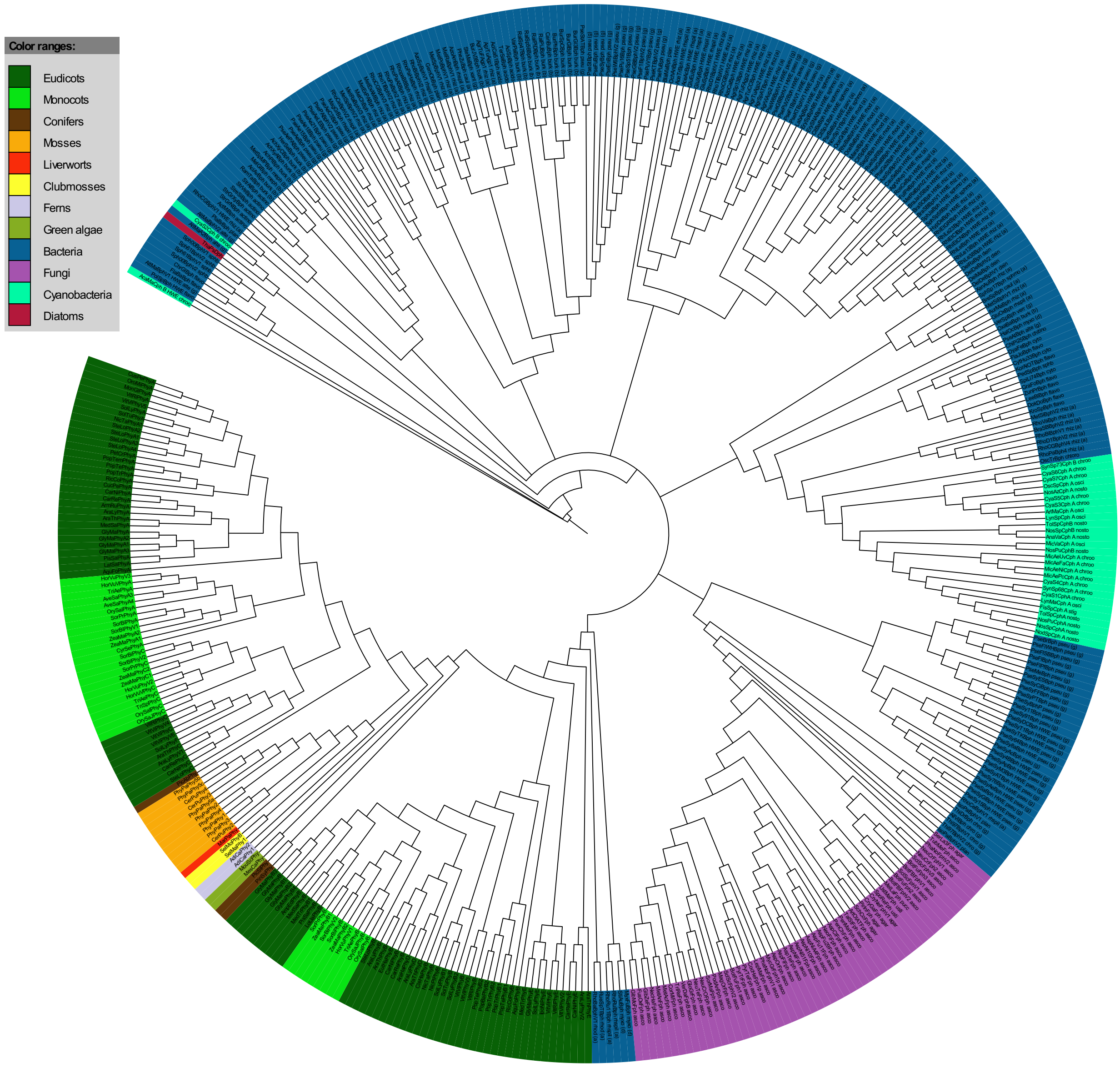
Color ranges:
Eudicots
Monocots
Conifers
Mosses
Liverworts
Clubmosses
Ferns
Green algae
Bacteria
Fungi
Cyanobacteria
Diatoms

P10

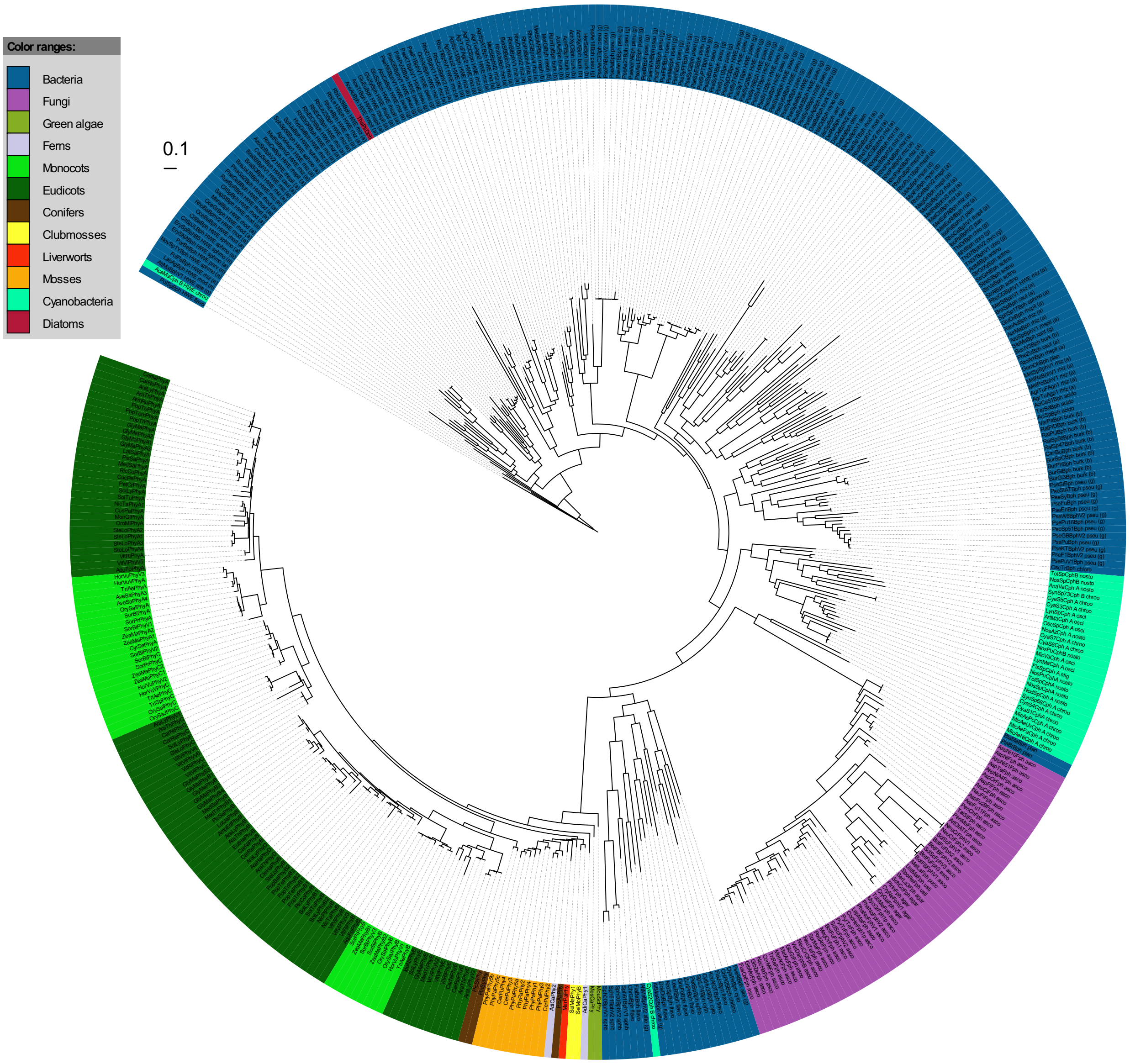
Color ranges:
Bacteria
Fungi
Green algae
Ferns
Monocots
Eudicots
Conifers
Clubmosses
Liverworts
Mosses
Cyanobacteria
Diatoms
0.1

P11

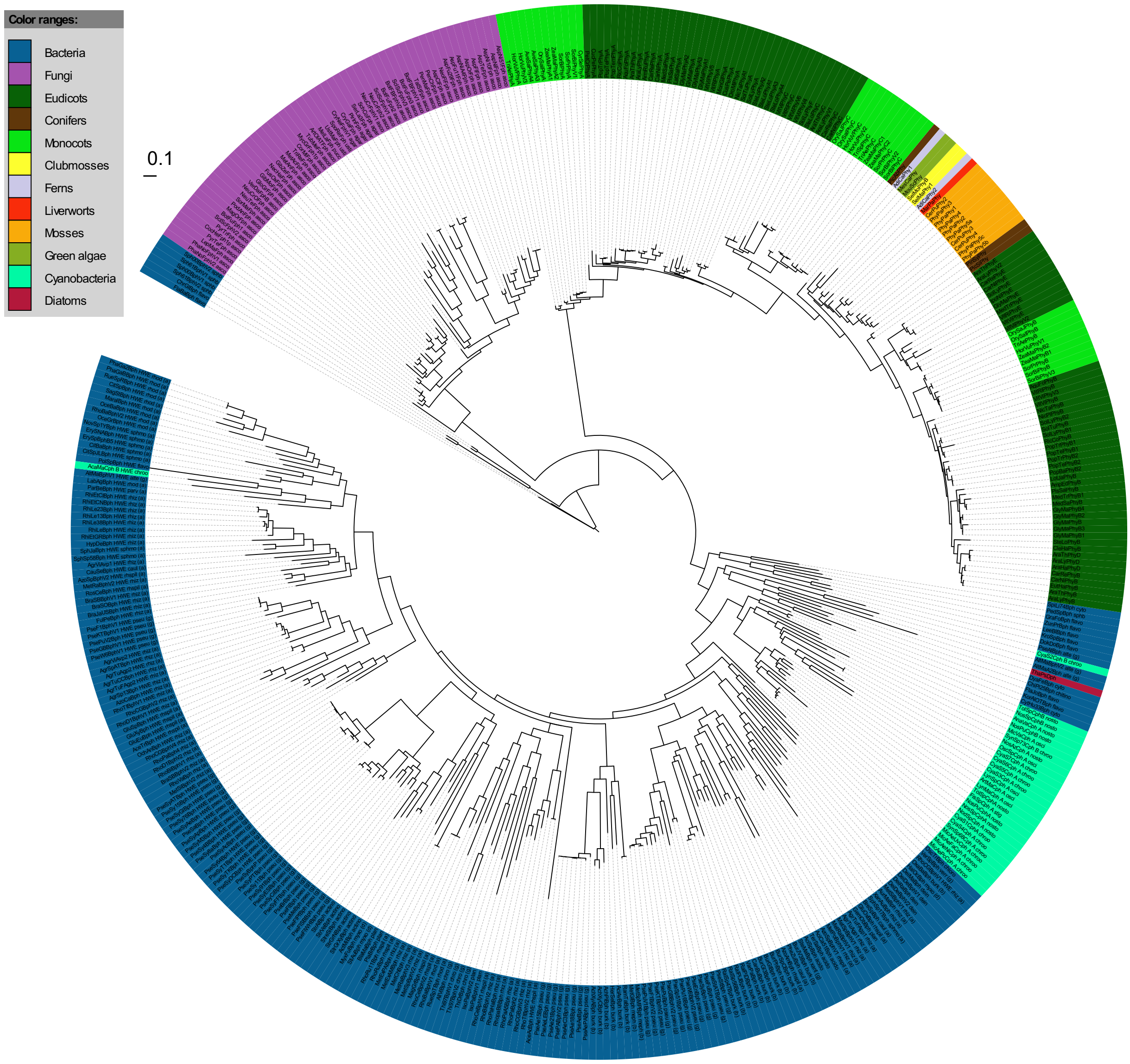
Color ranges:
Bacteria
Fungi
Eudicots
Conifers
Monocots
Clubmosses
Ferns
Liverworts
Mosses
Green algae
Cyanobacteria
Diatoms
0.1

P12

P13

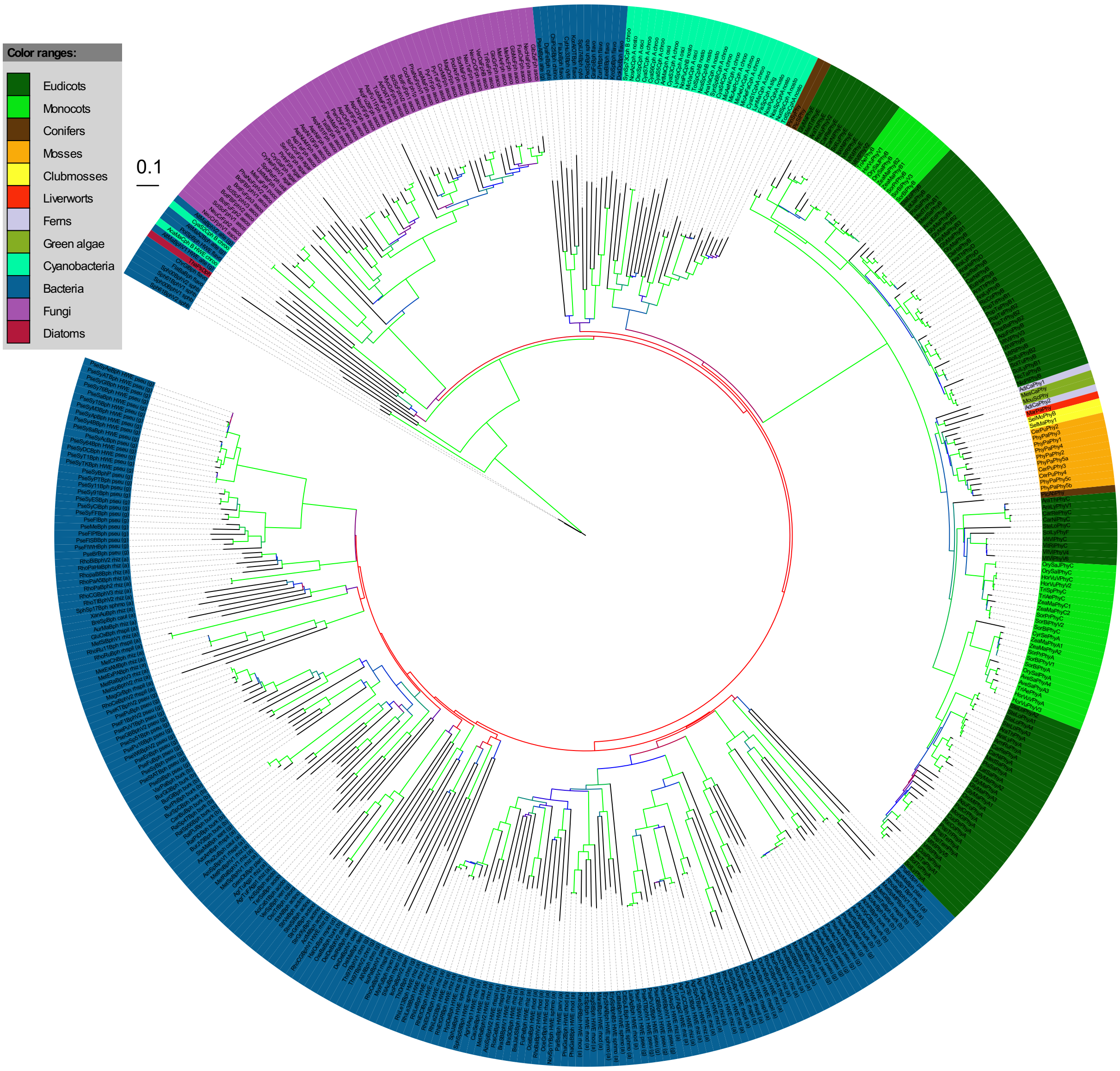

Color ranges:
Eudicots
Monocots
Conifers
Mosses
Clubmosses
Liverworts
Ferns
Green algae
Cyanobacteria
Bacteria
Fungi
Diatoms
0.1

P14

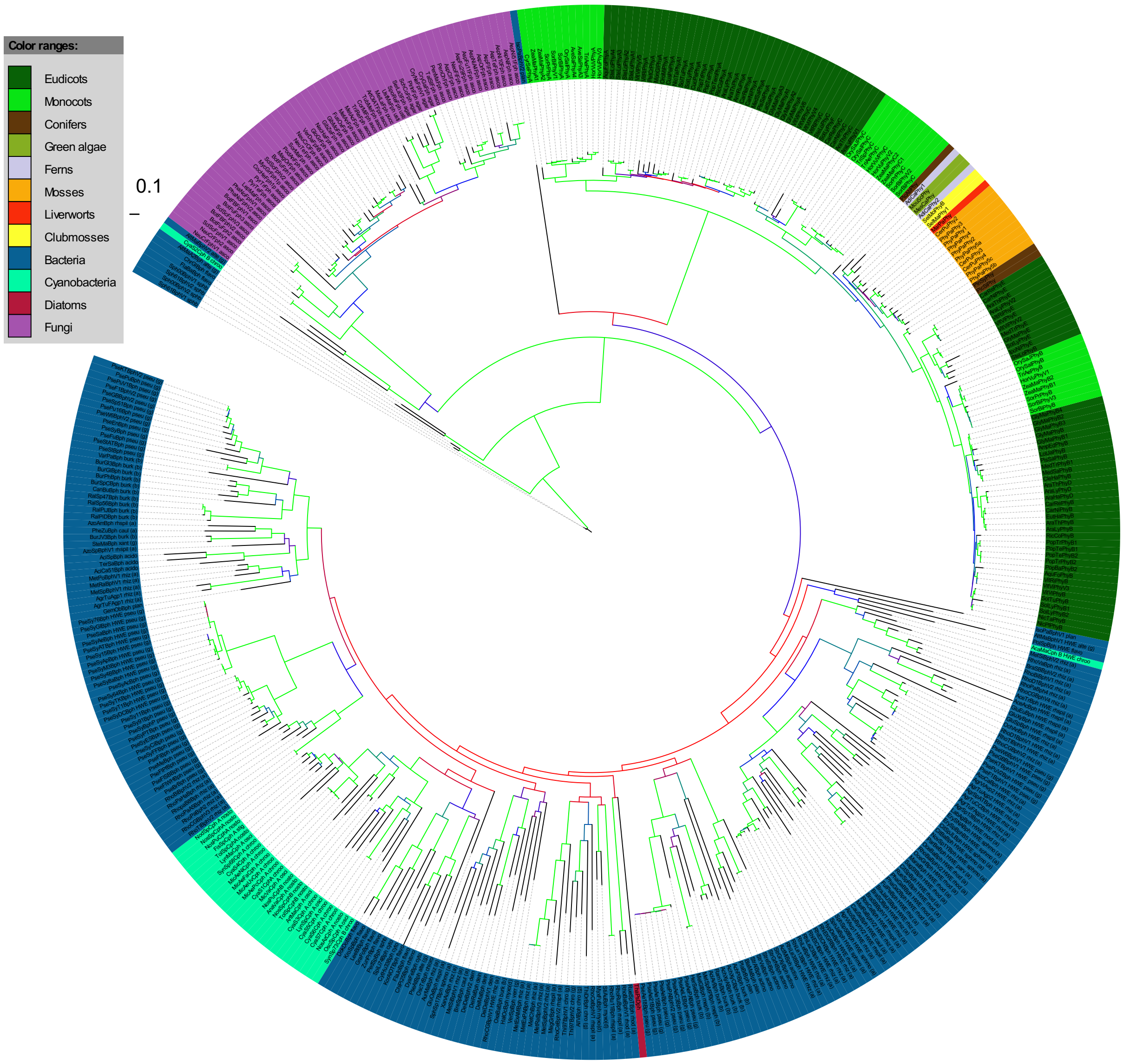
Color ranges:
Eudicots
Monocots
Conifers
Green algae
Ferns
Mosses
Liverworts
Clubmosses
Bacteria
Cyanobacteria
Diatoms
Fungi
0.1

P15

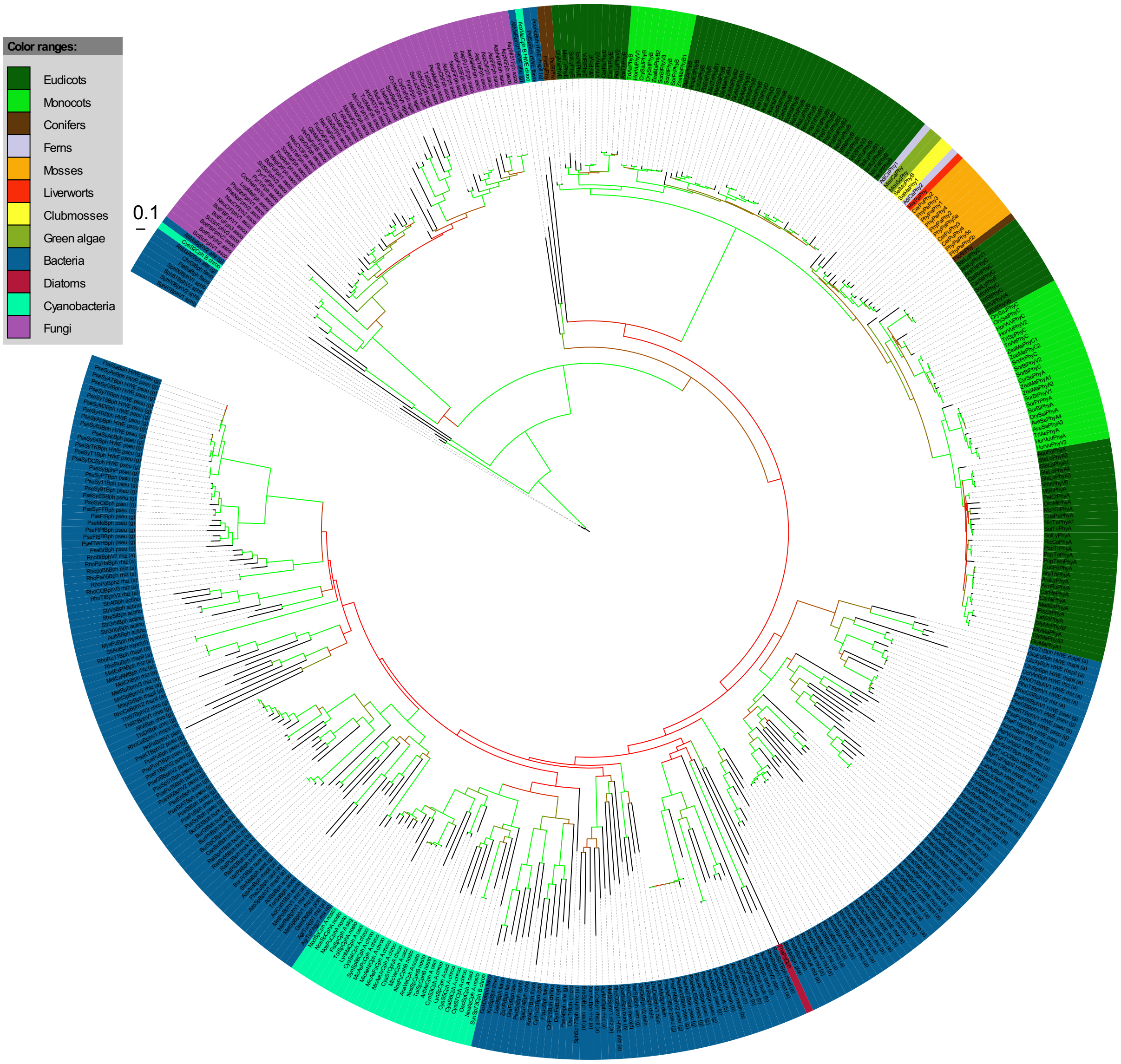
Color ranges:
Eudicots
Monocots
Conifers
Ferns
Mosses
Liverworts
Clubmosses
Green algae
Bacteria
Diatoms
Cyanobacteria
Fungi
0.1

P16

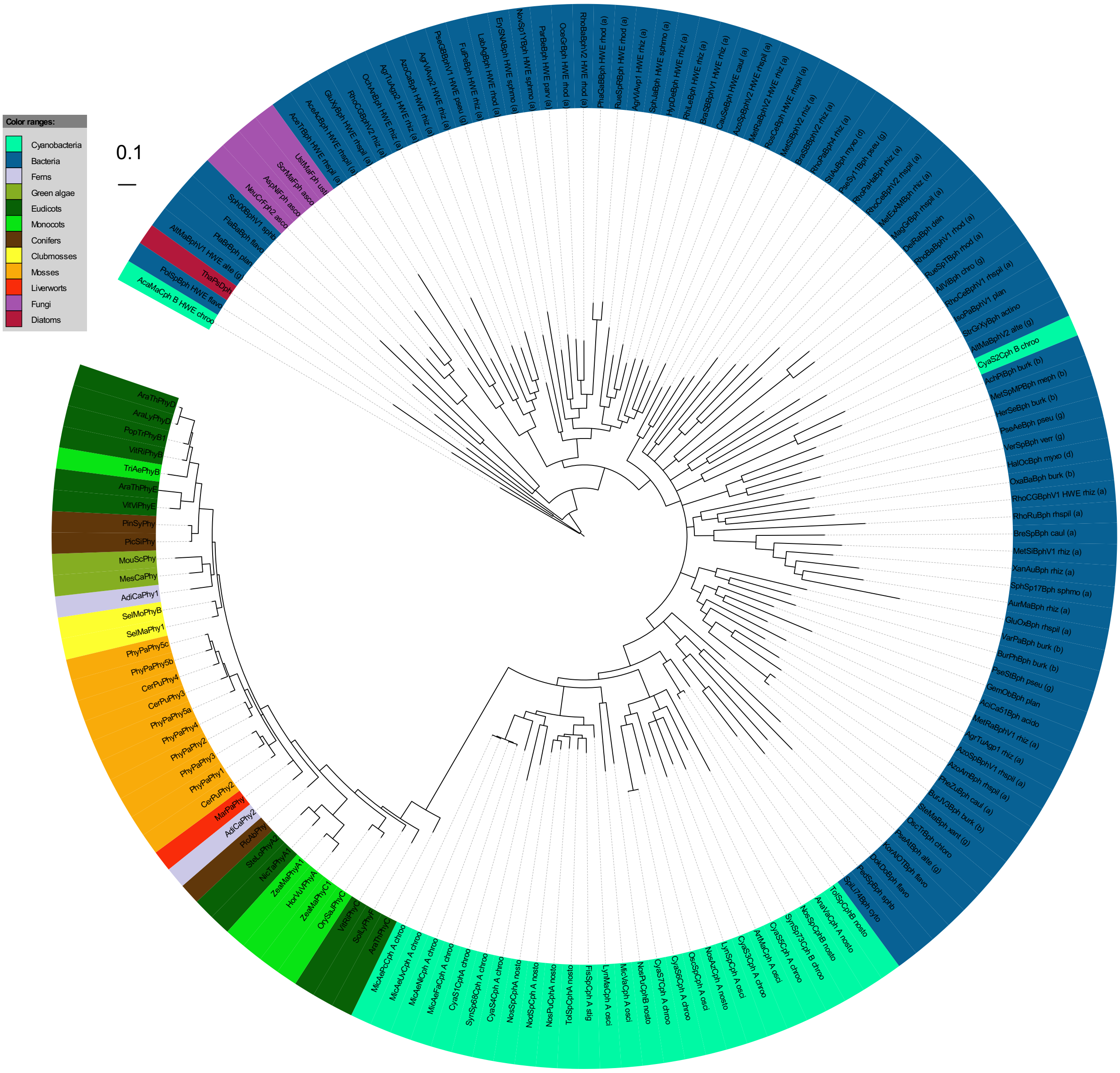

P17

Additional file 2. Phylogenetic PGP trees P1 to P17. The tree numbers refer to the summary in Table 1 . The color code is: blue for bacteria, light blue for cyanobacteria, orange for mosses, green for dicotyledonous plants, light green for monocotyledonous plants, brown for gymnosperms, red for liverworts, grey for ferns, purple for fungi and dark violet for diatom. Typically, phytochromes are denominated by a 5 letters species code together with an abbreviation for the particular group of phytochrome. Accession numbers and species / strain names are listed in supplemental Table 1. After each phytochrome abbreviation, additional taxonomic information is given for bacterial and fungal sequences: (a), α- proteobacterium; (b), ß-proteobacterium; (g), γ-proteobacterium; (d), δ-proteobacterium; acido, Adicobacteriales; actino, Actinomycetales; agar, Agaricomycetes (basidiomycetes); asco, Ascomycota; burk, Burkholderiales; caul, Caulobacterales; chitino, Chitinophagaceae; chroo, Chroococcales; cyto, Cytophagales; dein, Deinococcales; flavo, Flavobacteria; HWE, HWE histidine kinase; meph, Methylophilales; myxo, Myxococcales; nosto, Nostocales; osci, Oscillatoriales; parv, Parvulaculales; plan, Planctomycetales; pseu, Pseudomonadales; pucci, Pucciniomycotes (basidiomycetes); rhizo, Rhizobiales; rhod, Rhodobacterales; rhspil, Rhodospirillales; sphmo, Sphingomonadales; stig, Stigonematales; usti, Ustilagomycotina (basidiomycetes); verr, Verrucomicrobiales; xant, Xanthomonadales. The color of the branches indicate bootstrap values of 10-30% (red), 80-100% (green). Intermediate values are colored blue or with mixed colors. No information about branchlength is given in the PROTPARS trees P7, P8, P9 and P10.
