# Supplementary material for: Streptophyte phytochromes exhibit an N-terminus of cyanobacterial origin and a C-terminus of proteobacterial origin
Source: BMC Res Notes. 2015 Apr 13;8:144. doi: 10.1186/s13104-015-1082-3 (PMC4422448; doi:10.1186/s13104-015-1082-3)
Supplement: Additional file 6: — Phylogenetic trees constructed with PAS/PAS/HK of two streptophyte phytochromes and prokaryotic proteins. [file 13104_2015_1082_MOESM6_ESM.pdf]

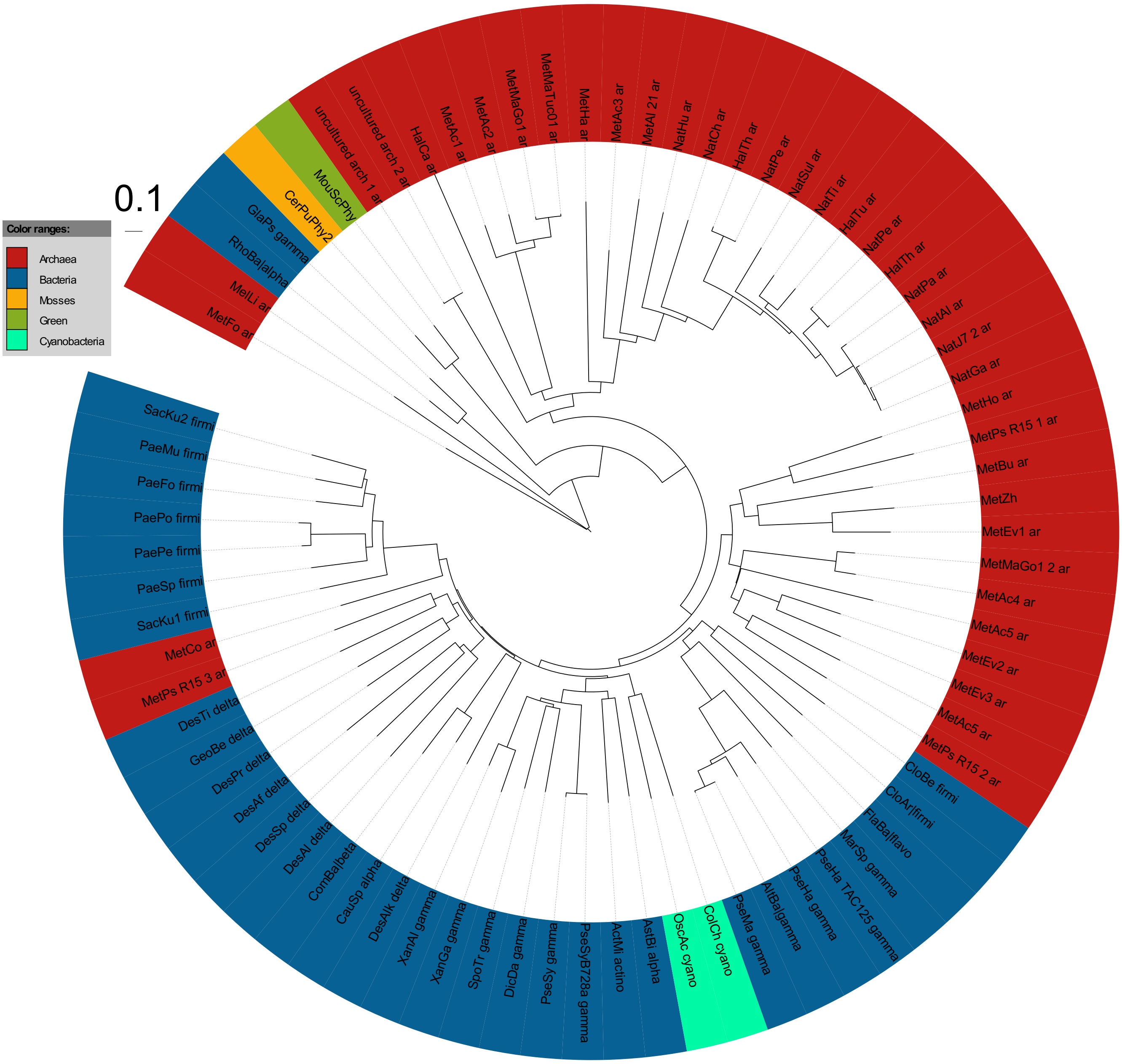

NEIGHBOR JOINING

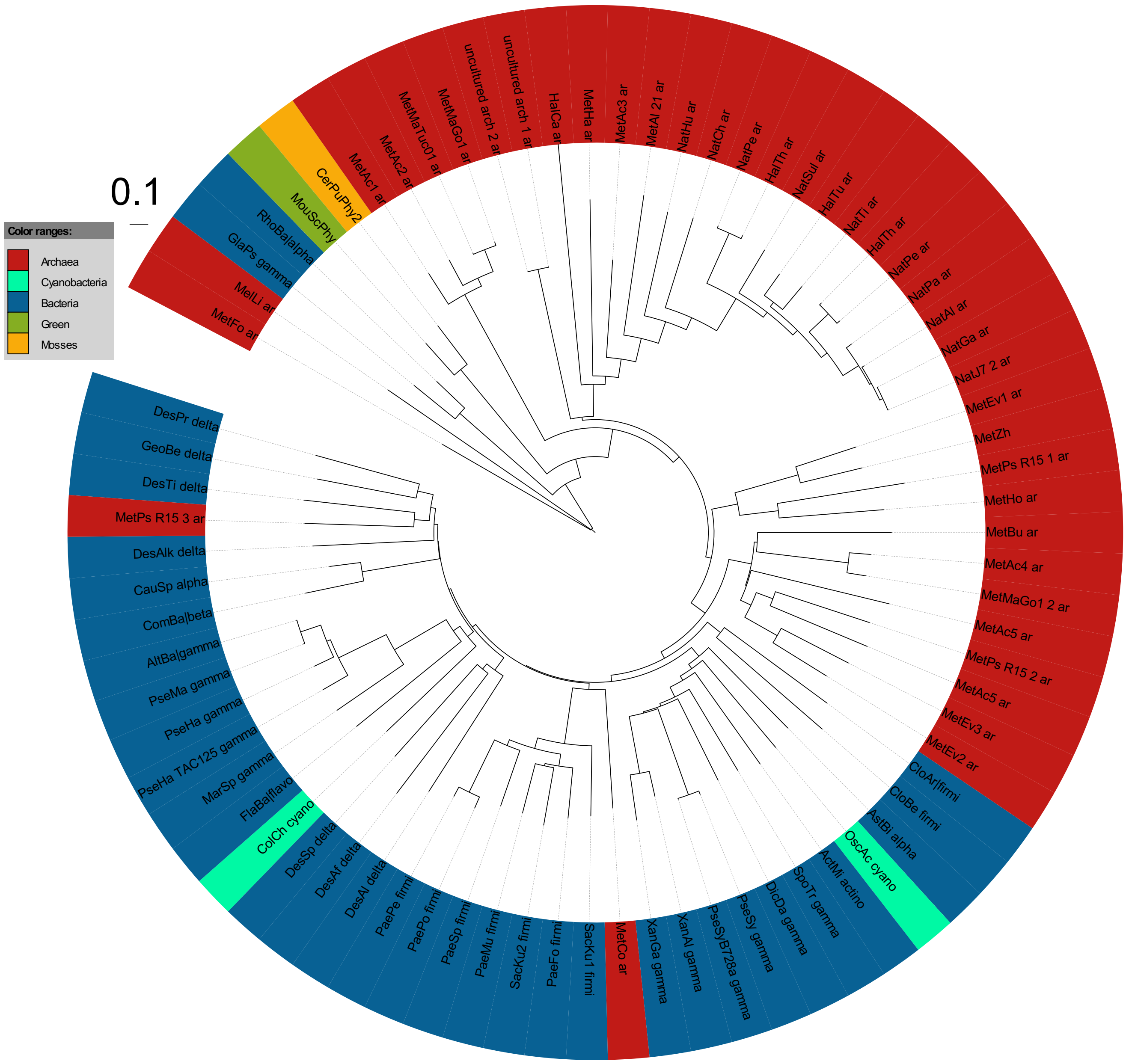
0.1
Color ranges:
Archaea
Cyanobacteria
Bacteria
Green
Mosses
MetFo ar
MelLi ar
GlaPs gamma
RhoBa|alpha
MouScPhy
CerPuPhy2
MetAc1 ar
MetAc2 ar
MetMaTuc01 ar
MetMaGo1 ar
uncultured arch 2 ar
uncultured arch 1 ar
HalCa ar
MetHa ar
MetAc3 ar
MetAl 21 ar
NatHu ar
NatCh ar
NatPe ar
HalTh ar
NatSul ar
HalTu ar
NatTi ar
HalTh ar
NatPe ar
NatPa ar
NatAl ar
NatGa ar
NatJ7 2 ar
MetEv1 ar
MetZh
MetPs R15 1 ar
MetHo ar
MetBu ar
MetAc4 ar
MetMaGo1 2 ar
MetAc5 ar
MetPs R15 2 ar
MetAc5 ar
MetEv3 ar
MetEv2 ar
CloAr|firmi
CloBe firmi
AstBi alpha
OscAc cyano
ActMi actino
SpoTr gamma
DicDa gamma
PseSy gamma
PseSyB728a gamma
XanAl gamma
XanGa gamma
MetCo ar
SacKu1 firmi
PaeFo firmi
SacKu2 firmi
PaeMu firmi
PaeSp firmi
PaePo firmi
PaePe firmi
DesAl delta
DesAf delta
DesSp delta
ColCh cyano
FlaBa|flavo
MarSp gamma
PseHa TAC125 gamma
PseHa gamma
PseMa gamma
AltBa|gamma
ComBa|beta
CauSp alpha
DesAlk delta
MetPs R15 3 ar
DesTi delta
GeoBe delta
DesPr delta

FITCH

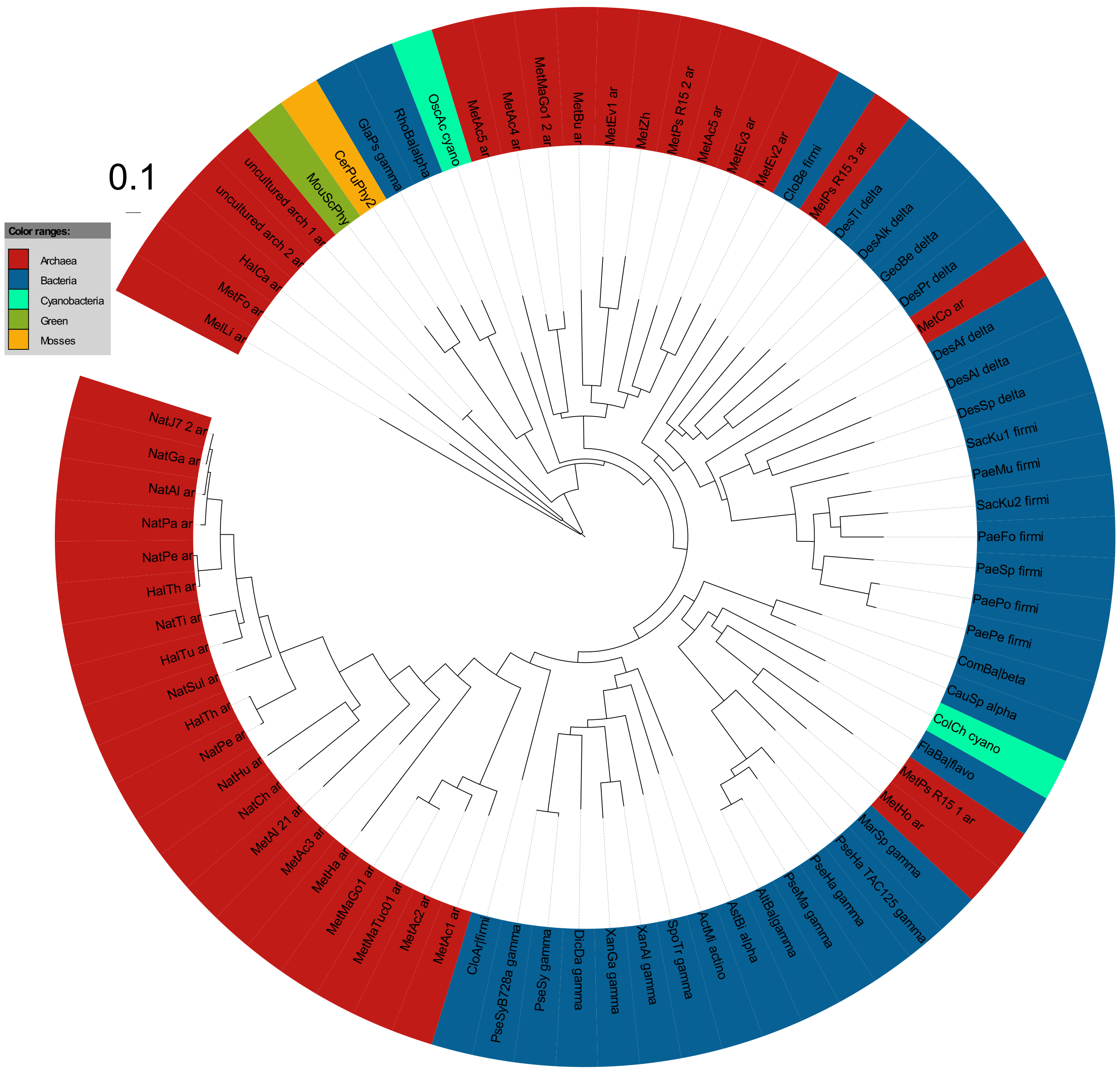
0.1
Color ranges:
Archaea
Bacteria
Cyanobacteria
Green
Mosses
MelLi ar
MetFo ar
HalCa ar
uncultured arch 2 ar
uncultured arch 1 ar
MouScPhy
CerPuPhy2
GlaPs gamma
RhoBalalpha
OscAc cyano
MetAc5 ar
MetAc4 ar
MetMaGo1 2 ar
MetBu ar
MetEv1 ar
MetZh
MetPs R15 2 ar
MetAc5 ar
MetEv3 ar
MetEv2 ar
CloBe firmi
MetPs R15 3 ar
DesTi delta
DesAlk delta
GeoBe delta
DesPr delta
MetCo ar
DesAf delta
DesAl delta
DesSp delta
SacKu1 firmi
PaeMu firmi
SacKu2 firmi
PaeFo firmi
PaeSp firmi
PaePo firmi
PaePe firmi
ComBalbeta
CauSp alpha
ColCh cyano
FlaBalflavo
MetPs R15 1 ar
MetHo ar
MarSp gamma
PseHa TAC125 gamma
PseHa gamma
PseMa gamma
AltBalgamma
AstBi alpha
ActMi actino
SpoTr gamma
XanAl gamma
XanGa gamma
DicDa gamma
PseSy gamma
PseSyB728a gamma
CloArfirmi
MetAc1 ar
MetAc2 ar
MetMaTuc01 ar
MetMaGo1 ar
MetHa ar
MetAc3 ar
MetAl 21 ar
NatCh ar
NatHu ar
NatPe ar
HalTh ar
NatSul ar
HalTu ar
NatTi ar
HalTh ar
NatPe ar
NatPa ar
NatAl ar
NatGa ar
NatJ7 2 ar

PROML

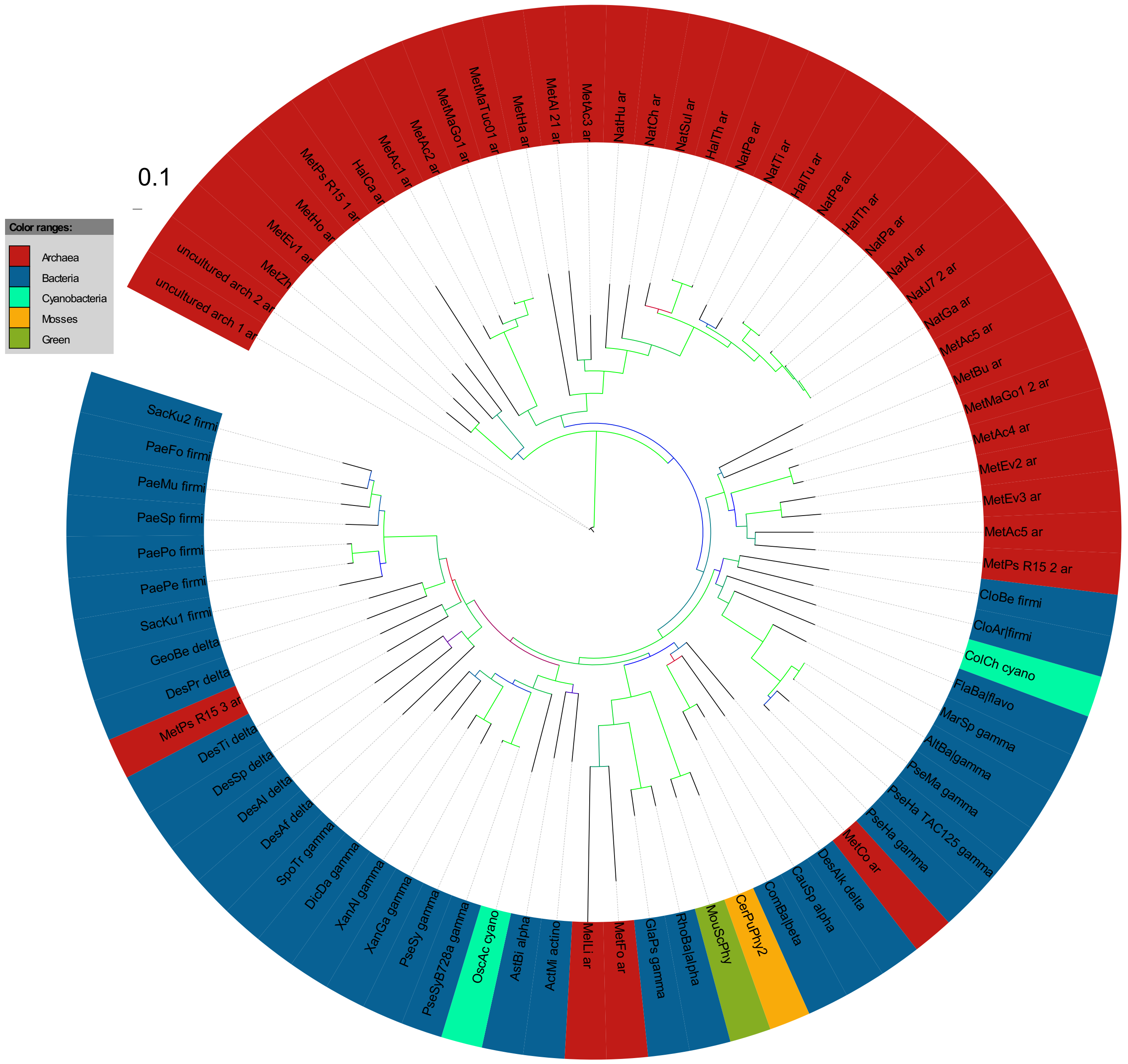
0.1
Color ranges:
Archaea
Bacteria
Cyanobacteria
Mosses
Green
uncultured arch 1 ar
uncultured arch 2 ar
MetZh
MetEv1 ar
MetHo ar
MetPs R15 1 ar
HalCa ar
MetAc1 ar
MetAc2 ar
MetMaGo1 ar
MetMaTuc01 ar
MetHa ar
MetAl 21 ar
MetAc3 ar
NatHu ar
NatCh ar
NatSul ar
HalTh ar
NatPe ar
NatTi ar
HalTu ar
NatPe ar
HalTh ar
NatPa ar
NatAl ar
NatJ7 2 ar
NatGa ar
MetAc5 ar
MetBu ar
MetMaGo1 2 ar
MetAc4 ar
MetEv2 ar
MetEv3 ar
MetAc5 ar
MetPs R15 2 ar
CloBe firmi
CloAr|firmi
ColCh cyano
FlaBa|flavo
MarSp gamma
AltBa|gamma
PseMa gamma
PseHa TAC125 gamma
PseHa gamma
MetCo ar
DesAlk delta
CauSp alpha
ComBa|beta
CerPuPhy2
MouScPhy
RhoBa|alpha
GlaPs gamma
MetFo ar
MelLi ar
ActMi actino
AstBi alpha
OscAc cyano
PseSyB728a gamma
PseSy gamma
XanGa gamma
XanAl gamma
DicDa gamma
SpoTr gamma
DesAf delta
DesAl delta
DesSp delta
DesTi delta
MetPs R15 3 ar
DesPr delta
GeoBe delta
SacKu1 firmi
PaePe firmi
PaePo firmi
PaeSp firmi
PaeMu firmi
PaeFo firmi
SacKu2 firmi

PHYML

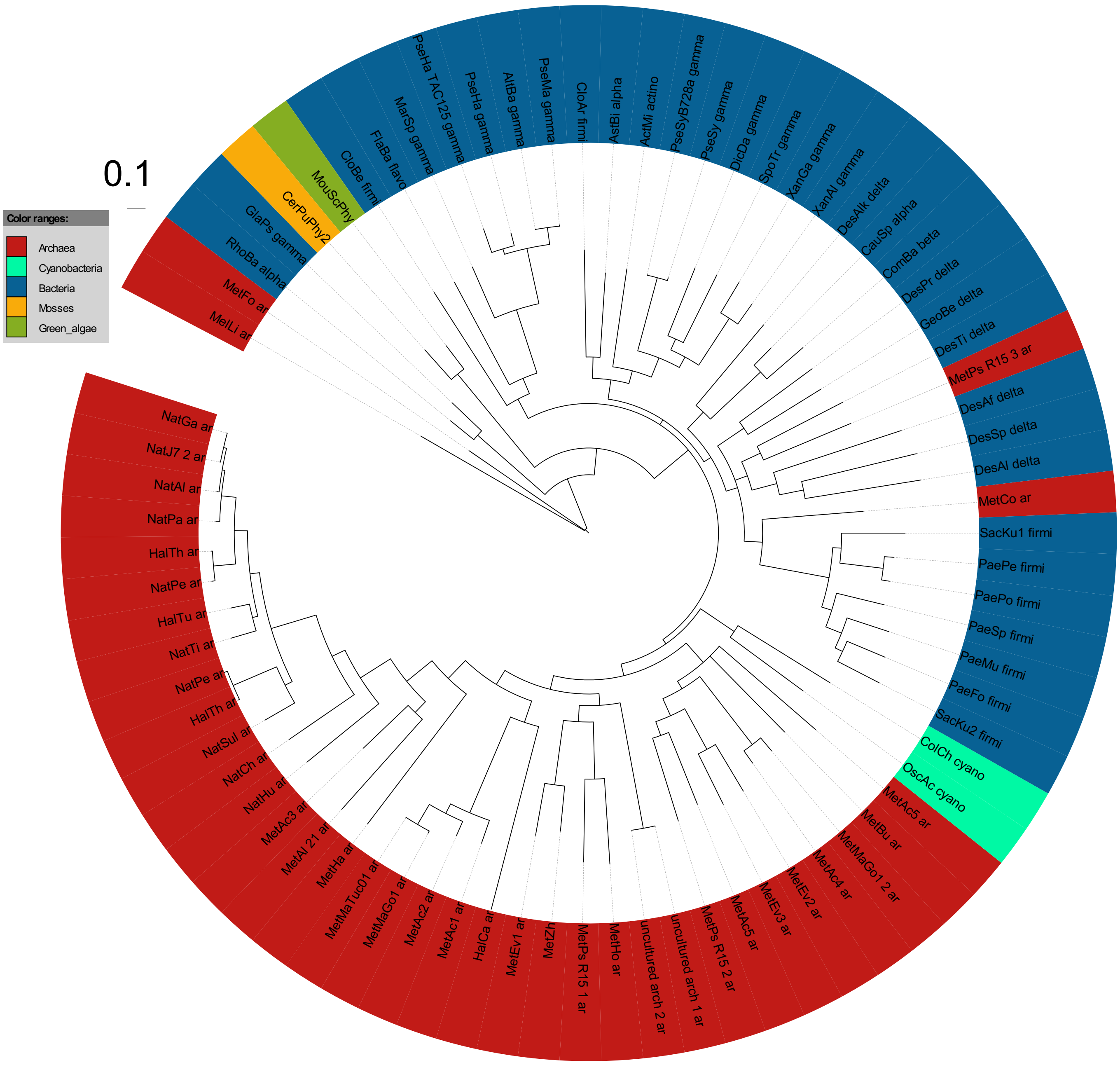

MrBayes

Additional File 3. Phylogenetic trees constructed with PAS/PAS/HK of two streptophyte phytochromes and prokaryotic proteins. Alignment was performed with MUSCLE and phylogenetic programs were run with parameters as in Table 1 (no bootstrapping). PHYML was run with the LG matrix and aRLT. Green lines indicate 80-100% aRLT values, blue lines 50-80%, 40-50% are in magenta. Red indicates archaeal sequences, blue and light blue bacterial and cyanobacterial sequences, respectively. Moss and algal phytochromes are in orange and brown, respectively (see Supplemental Table 1 for accessions). Abbreviations are: ar, archaebacterial; alpha, α-proteobacterium; beta, β-proteobacterium; delta, δ-proteobacterium; gamma, γ-proteobacterium; firmi, firmicutes. Species names are abbreviated by the 5 letter code. The following list gives the GI numbers (first field) and NCBI reference number (second field) of the prokaryotic proteins.

```
MelLi_ar ar_490178053_ref_WP_004076678.
HalCa_ar ar_494345671_ref_WP_007188961.
MetFo_ar ar_432329823_ref_YP_007247966.
MetAc1_ar ar_20089101_ref_NP_615176.
MetAc2_ar ar_20089441_ref_NP_615516.
MetMaGo1_ar ar_21226270_ref_NP_632192.
MetMaTuc01_ar ar_452208790_ref_YP_007488904.
MetHo_ar ar_435851282_ref_YP_007312868.
MetHa_ar ar_386002448_ref_YP_005920747.
uncultured_arch_1_ar ar_268325194_emb_CBH38782.
uncultured_arch_2_ar ar_268326254_emb_CBH39842.
NatHu_ar ar_493703449_ref_WP_006653182.
NatCh_ar ar_493159450_ref_WP_006167331.
HalTh_ar ar_493700043_ref_WP_006649833.
NatPe_ar ar_433592754_ref_YP_007282250.
NatSul_ar ar_495438327_ref_WP_008163022.
NatPe_ar ar_433591829_ref_YP_007281325.
HalTh_ar ar_493699167_ref_WP_006648974.
NatPa_ar ar_493196572_ref_WP_006186732.
NatAl_ar ar_494168714_ref_WP_007108443.
NatJ7_2_ar ar_397775084_ref_YP_006542630.
```

```
NatGa_ar ar_495734402_ref_WP_008458981.
NatTi_ar ar_493009759_ref_WP_006089203.
HalTu_ar ar_284164195_ref_YP_003402474.
MetAl_21_ar ar_325958983_ref_YP_004290449.
MetAc3_ar ar_20090134_ref_NP_616209.
MetMaGo1_2_ar ar_21228875_ref_NP_634797.
MetAc4_ar ar_20092182_ref_NP_618257.
MetPs_R15_1_ar ar_410671421_ref_YP_006923792.
MetZh ar_336476833_ref_YP_004615974.
MetEv1_ar ar_298675480_ref_YP_003727230.
MetAc5_ar ar_20090015_ref_NP_616090.
MetBu_ar ar_91772955_ref_YP_565647.
MetEv2_ar ar_298676132_ref_YP_003727881.
MetEv3_ar ar_298674344_ref_YP_003726094.
MetAc5_ar ar_20091132_ref_NP_617207.
MetPs_R15_2_ar ar_410671838_ref_YP_006924209.
MetCo_ar ar_330507467_ref_YP_004383895.
MetPs_R15_3_ar ar_410672020_ref_YP_006924391.
RhoBa_alpha ba495463062_ref_WP_008187754.
GlaPs_gamma ba476415902_ref_YP_007545820.
FlaBa_flavo ba495529611_ref_WP_008254239.
CloBe_firmi ba150017917_ref_YP_001310171.
DesSp_delta ba494790607_ref_WP_007526015.
CloAr_firmi ba497924816_ref_WP_010238972.
ColCh_cyano ba493030371_ref_WP_006099772.
OscAc_cyano ba428213830_ref_YP_007086974.
DesAl_delta ba527043629_ref_WP_020886803.
DesAf_delta ba492833326_ref_WP_005987280.
DesPr_delta ba320353300_ref_YP_004194639.
GeoBe_delta ba197117615_ref_YP_002138042.
MarSp_gamma ba152995118_ref_YP_001339953.
PseHa_TAC125_gamma ba77360094_ref_YP_339669.
PseHa_gamma ba489051230_ref_WP_002961437.
AltBa_gamma ba493845295_ref_WP_006792352.
PseMa_gamma ba498243408_ref_WP_010557564.
AstBi_aslpha ba493315350_ref_WP_006272752.
SacKu1_firmi ba517807288_ref_WP_018977496.
PaePe_firmi ba498033380_ref_WP_010347536.
PaePo_firmi ba308067783_ref_YP_003869388.
PaeSp_firmi ba516424588_ref_WP_017813986.
PaeMu_firmi ba511629456_ref_YP_006193285.2.
PaeFo_firmi ba518468174_ref_WP_019638381.
SacKu2_firmi ba517808225_ref_WP_018978433.
CauSp_alpha ba167644688_ref_YP_001682351.
ComBa_beta ba550991503_ref_YP_008682113.
DicDa_gamma ba242240530_ref_YP_002988711.
PseSyB728a_gamma ba66046171_ref_YP_236012.
PseSy_gamma ba489506315_ref_WP_003411190.
SpoTr_gamma ba551338691_ref_WP_022958111.
XanGa_gamma ba493495090_ref_WP_006449713.
XanAl_gamma ba285018741_ref_YP_003376452.
ActMi_actino ba383777032_ref_YP_005461598.
DesAlk_delta ba218782287_ref_YP_002433605.
DesTi_delta ba392410312_ref_YP_006446919.
```
